# Lung-resident SARS-CoV-2 peptide-specific immune responses in perfused 3D human lung explant models

**DOI:** 10.3389/fbioe.2025.1587080

**Published:** 2025-07-08

**Authors:** Kayla F. Goliwas, Anthony M. Wood, Christopher S. Simmons, Rabisa Khan, Saad A. Khan, Yong Wang, Rekha Ramachandran, Joel L. Berry, Mohammad Athar, James A. Mobley, Young-il Kim, Victor J. Thannickal, Kevin S. Harrod, James M. Donahue, Jessy S. Deshane

**Affiliations:** ^1^ Department of Medicine, Division of Pulmonary, Allergy, and Critical Care Medicine, University of Alabama at Birmingham, Birmingham, AL, United States; ^2^ Department of Medicine, Division of Preventative Medicine, University of Alabama at Birmingham, Birmingham, AL, United States; ^3^ Department of Biomedical Engineering, University of Alabama at Birmingham, Birmingham, AL, United States; ^4^ Department of Dermatology, University of Alabama at Birmingham, Birmingham, AL, United States; ^5^ Department of Anesthesiology and Perioperative Medicine, University of Alabama at Birmingham, Birmingham, AL, United States; ^6^ John W. Deming Department of Medicine, Tulane University School of Medicine and Southeast Veterans Healthcare System, New Orleans, LA, United States; ^7^ Department of Surgery, University of Alabama at Birmingham, Birmingham, AL, United States

**Keywords:** human lung-tissue model, SARS-CoV-2 infection, COVID-19, perfused lung explant, local antiviral immune response

## Abstract

**Introduction:**

Multi-specific and long-lasting T-cell immunity has been recognized to indicate long-term protection against pathogens, including the novel coronavirus, SARS-CoV-2, which is the causative agent of the COVID-19 pandemic. Functional significance of peripheral memory T cells in individuals recovered from COVID-19 (COVID-19^+^) is beginning to be appreciated; however, the role of lung tissue-resident memory (lung TRM) T cells in SARS-CoV-2 infection is still being investigated. This is, in part, due to the lack of preclinical tissue models available to follow the convalescence period.

**Methods:**

Here, we utilize a perfused three-dimensional (3D) human lung-tissue model and show pre-existing local T-cell immunity against SARS-CoV-2 proteins in lung tissues.

**Results:**

We report *ex vivo* maintenance of functional multi-specific IFN-γ-secreting lung TRM T cells in COVID-19^+^ and their induction in lung tissues of vaccinated COVID-19^+^ subjects. Importantly, we identify SARS-CoV-2 peptide-responding memory B cells and IgA^+^ plasma cells in *ex vivo* cultured lung tissues of COVID-19^+^. Furthermore, lung tissue IgA levels were increased in COVID-19^+^ and responded to peptide stimulation.

**Discussion:**

In our study, we highlight the importance of utilization of human lung-tissue models to understand the local antiviral immune response in the lung to protect against SARS-CoV-2 infection.

## Introduction

COVID-19, the disease caused by the novel coronavirus SARS-CoV-2, is a global health concern (Centers for Disease Control and Prevention, 2022; BioSpace, 2020). Infected individuals develop lymphopenia and demonstrate hyperactivated and exhausted T-cell responses that contribute to prolonged immune dysregulation, which is a hallmark of SARS-CoV-2 infection (De Biasi et al., 2020; Le Bert et al., 2020; de Candia et al., 2021; Files et al., 2021; Toor et al., 2021). Although vaccine efforts are successful, the emergence of variants (Farinholt et al., 2021; Olsen et al., 2021), persistence of infection, and vaccine hesitancy continue to pose problems for eradication and management of COVID-19. All approved COVID-19 vaccines, to date, utilize intramuscular delivery of SARS-CoV-2 spike mRNA or inactivated virus to elicit humoral immunity, a strategy that has not offered long-term protection.

An important determinant for recovery and long-term protection is coronavirus-specific T-cell immunity (Zhao et al., 2010; Channappanavar et al., 2014; Zhao et al., 2016). During the initial phase of the pandemic, 20%–50% of unexposed individuals showed T-cell reactivity to SARS-CoV-2 antigen peptide pools (Grifoni et al., 2020a; Grifoni et al., 2020b; Le Bert et al., 2020; Files et al., 2021; Nelde et al., 2021; Schulien et al., 2021; Diniz et al., 2022; Swadling et al., 2022); the presence of SARS-CoV-2-specfic T cells in individuals with no prior history of SARS suggests pre-existing cross-reactive immune memory to seasonal coronaviruses (Le Bert et al., 2020; Niessl et al., 2021; Schulien et al., 2021; Kundu et al., 2022). In the majority of COVID-19-recovering individuals (COVID-19^+^), larger overall SARS-CoV-2-specific CD4^+^ and CD8^+^ T-cell responses were observed with severe disease, yet an increase in polyfunctional CD8^+^ T cells was noted in mild cases (Kared et al., 2020; Rydyznski Moderbacher et al., 2020; Sattler et al., 2020). As memory T-cell responses induced by previous viral pathogens can shape susceptibility to subsequent viral infections, including SARS-CoV-2, and/or influence clinical severity of COVID-19, pre-existing memory T cells that recognize SARS-CoV-2 have been implicated in a more robust response to SARS-CoV-2 (Lipsitch et al., 2020; Ni et al., 2020; Peng et al., 2020; Rydyznski Moderbacher et al., 2020; Jung et al., 2021; Rodda et al., 2021; Tavukcuoglu et al., 2021; Le Bert et al., 2020). An increase in memory CD4^+^ and CD8^+^ T cells may enable direct antiviral immunity in the lungs and nasopharynx, with pre-existing CD4^+^ memory T cells potentially influencing vaccination outcomes, leading to a quicker and more robust immune response and development of neutralizing antibodies (Grigoryan and Pulendran, 2020). Alternatively, pre-existing immunity could be detrimental due to antibody-mediated inflammation or inadequate immune responses (Sette and Crotty, 2020). The assessment of the SARS-CoV-2-reactive T-cell pool including lung tissue-resident memory (TRM) T cells has been challenging; circulating T cells may not represent lung-specific responses to viral infection or reflect direct antiviral immunity in the lungs or the nasopharynx, and very few studies have evaluated the overall SARS-CoV-2-specific T-cell responses in the lungs (Mathew et al., 2020; Meckiff et al., 2020).

In addition to T-cell immunity, the most anticipated and monitored protective response is the evolution of antibody immunity to SARS-CoV-2 (Gaebler et al., 2021). Waning antibody levels after SARS-CoV-2 infection (Abbasi, 2021; Levin et al., 2021; Tre-Hardy et al., 2021a; Tre-Hardy et al., 2021b) and reappearance after SARS-CoV-2 vaccination have been reported (Cagigi et al., 2021; Havervall et al., 2022; Sheikh-Mohamed et al., 2022). Understanding the memory B-cell response and the presence and durability of antibodies in the local lung-tissue microenvironment will provide insights into the ability of infected individuals to neutralize the virus locally and prevent virus spread; very few studies have addressed such localized responses (Gaebler et al., 2021). Early SARS-CoV-2-specific humoral responses were dominated by IgA and not IgG antibodies (Sterlin et al., 2021; Wang et al., 2021); however, their presence and function in the lung tissue of COVID-19^+^ are largely unknown.

Although lung alveolar, airway, and bronchial organoids have been widely used to monitor viral infection and identify putative therapeutics, state-of-the-art human tissue-derived models are needed to identify immune changes in response to infection, as most organoids lack maintenance of the immune component (Han et al., 2022). Models that maintain the native tissue architecture and diverse cellular composition are needed to fully comprehend local alterations that result from infection. Herein, we utilize a perfused three-dimensional (3D) lung-tissue culture model that maintains the tissue architecture and diverse immune cell composition of the human lung for the assessment of pre-existing T-cell immunity and memory B-cell responses in lung tissues of previously uninfected (UN) and COVID-19 convalescent (COVID-19^+^) individuals. We demonstrate the presence of pre-existing T-cell immunity in UN individuals and SARS-CoV-2 peptide-responding hyperactivated T cells in COVID-19^+^. Furthermore, we identify functional lung TRM cells in early convalescing COVID-19^+^, SARS-CoV-2 peptide-responder TRM cells in vaccinated COVID-19^+^, and memory B-cell response in the lungs of COVID-19^+^. Importantly, we are able to quantitate IgA levels and IgA-secreting plasma cells in the lungs of COVID-19^+^. Together, this suggests pre-existing immunity in the lungs of UN individuals and boosted immunity in the lungs of COVID-19^+^ individuals.

## Results

### *Ex vivo* perfusion of human lung tissues

**FIGURE 1:**
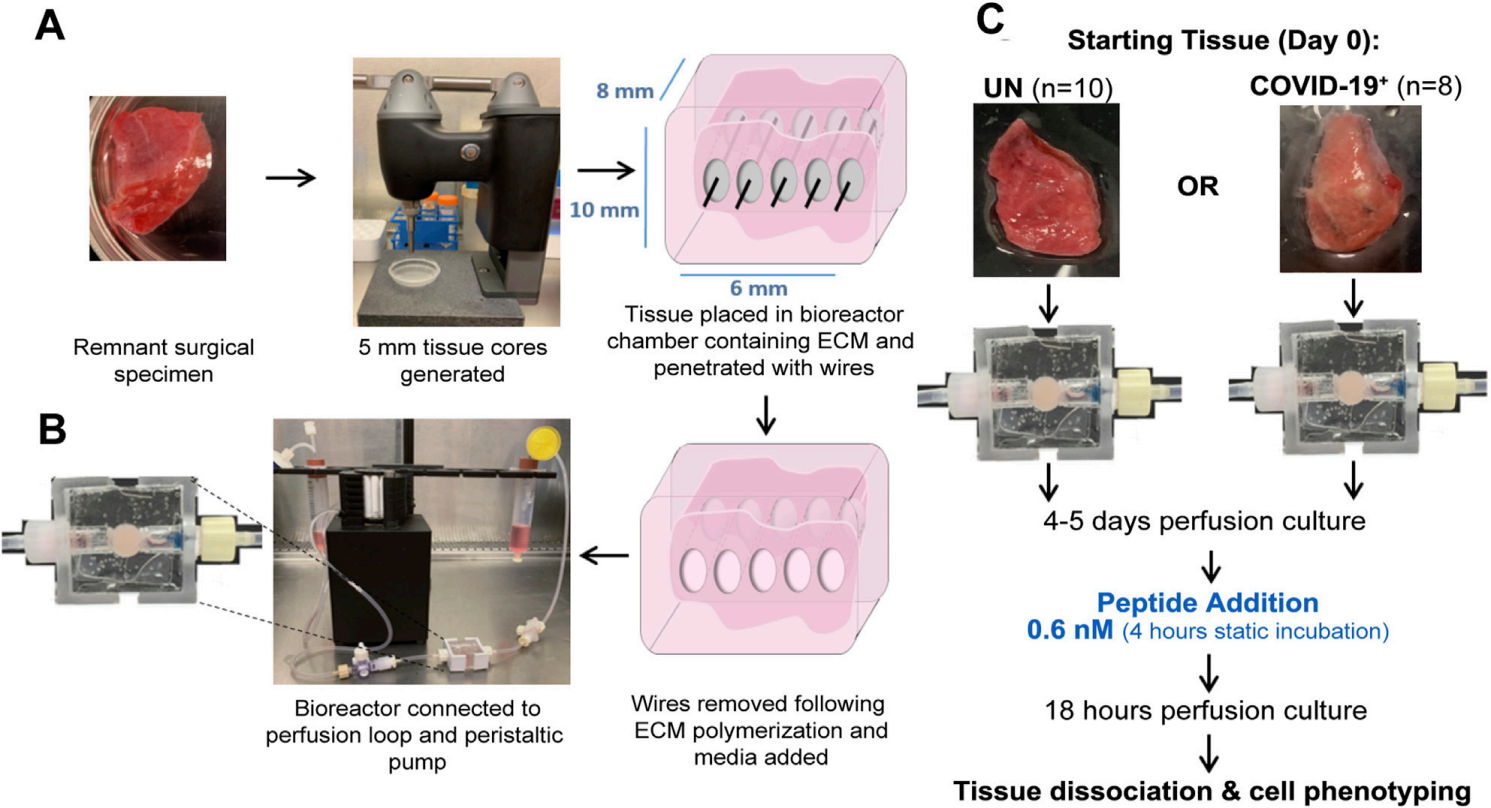
*Ex vivo* perfusion culture of the human lung tissue and exposure to SARS-CoV-2 peptides. **(A)** *Ex vivo* model setup process. **(B)** Bioreactor chamber showing ECM volume-containing tissue. **(C)** Peptide exposure protocol.

Remnant surgical specimens from individuals (no history of SARS-CoV-2 infection) undergoing lung resection surgeries were collected. For *ex vivo* culture, 5-mm-diameter tissue cores were generated, and one tissue core was placed into the central chamber of a bioreactor containing a mixture of extracellular matrix (ECM) components. The tissue/ECM support was penetrated with five 400-micron Teflon-coated stainless steel wires to generate through-channels for adequate tissue perfusion. Wires were removed following ECM polymerization. A serum-free, defined tissue culture media was then perfused from a media reservoir through the tissue volume and collected in a collection reservoir using a peristaltic pump (Figures 1A,B). Using this culture system, we observed maintenance of the histologic tissue architecture (Supplementary Figure S1A) and cell density (cells/area, Supplementary Figure S1B) over a 2-week culture period. Furthermore, lactate dehydrogenase (LDH) remained unchanged during culture (Supplementary Figure S1C), indicating sustained viability. The ECM composition utilized here has been used with earlier prototype bioreactors to generate viable cell culture models of lung and breast carcinoma (Goliwas et al., 2016; Goliwas et al., 2017; Goliwas et al., 2021). These platforms were adapted here for the *ex vivo* culture of human lung tissues. Cell phenotyping via flow cytometry showed the maintenance of lung epithelial and endothelial cells, along with fibroblasts and lymphocytes including CD8^+^ T cells (Supplementary Figure S1D–H), and proteomic analyses showed the maintenance of the human lung ECM (Supplementary Figure S1I).

### Cellular landscape and response to SARS-CoV-2 peptides within the lung tissues of previously uninfected and convalescent individuals

Local immune responses to SARS-CoV-2 peptides were compared in lung-tissue specimens collected from 10 individuals who were not previously infected with SARS-CoV-2 (UN) and eight convalescent individuals who previously tested positive for SARS-CoV-2 and cleared the virus (COVID-19^+^, Supplementary Tables S1,S2 with subject demographics). Tissue cores were cultured *ex vivo* using the bioreactor platform. Following 4–5 days of perfusion culture, peptide pools covering the SARS-CoV-2 membrane glycoprotein (M-peptide), nucleocapsid phosphoprotein (N-peptide), or the immunodominant sequence of the spike protein (S-peptide) were added to circulating media; cellular responses were then compared to those in vehicle control-exposed tissues (Figure 1C). Minimal cell apoptosis and necrosis were noted in the perfused cultures (Supplementary Figure S2A,B), indicating minimal loss in tissue viability.

**FIGURE 2:**
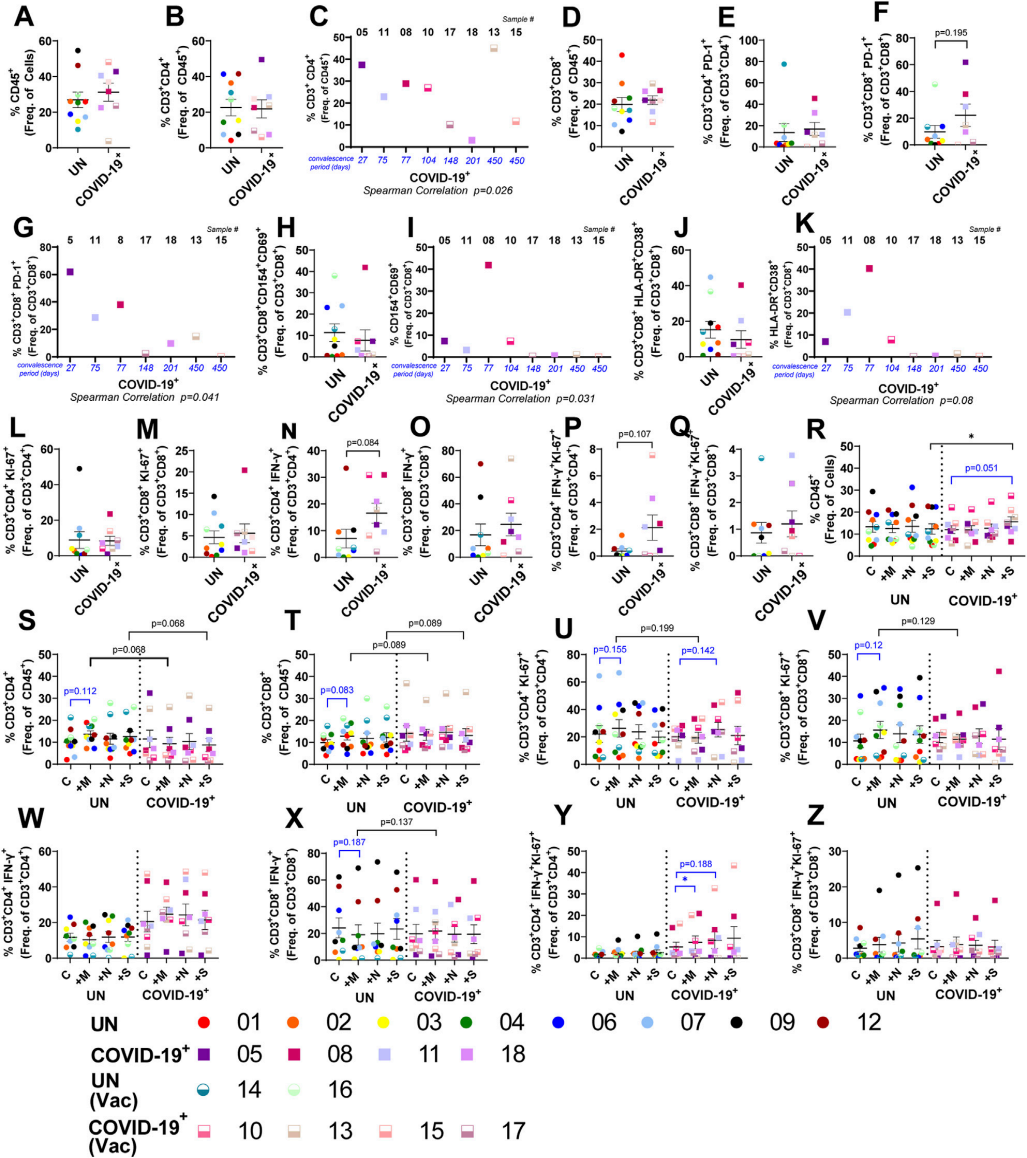
Local T-cell response to SARS-CoV-2 peptides within lung tissue cores. **(A**, **B**, **D–F**, **H**, **J**, and **L–Q)** Baseline T-cell differences within lung tissue from uninfected (UN) individuals and individuals recovered from COVID-19 (COVID-19^+^). **(C**, **G**, **I**, and **K)** Correlation between the convalescence period for COVID-19^+^ samples and the percentage of CD4^+^ T cells **(C)**, PD-1^+^ CD8^+^ T cells **(G)**, CD154^+^ CD69^+^ CD8^+^ T cells **(J)**, and HLA-DR^+^ CD38^+^ CD8^+^ T cells **(L)**. **(R–Z)** Impact of SARS-CoV-2 peptide exposure on T-cell populations in UN and COVID-19^+^ lung tissues (n = 10 UN and n = 8 COVID-19^+^ [mean (center line) ± SEM]). Statistics shown in blue are comparisons between control and peptide-exposed samples within each group (UN and COVID-19^+^). Statistics shown in black are the change in response between UN and COVID-19^+^ for each peptide when compared to the corresponding control.

Baseline phenotyping showed no difference in epithelial cell populations, endothelial cells, CD45^+^ immune cells, TNF-α^+^ immune cells, CD4^+^ T cells, or CD8^+^ T cells within the lung tissues of COVID-19^+^ and UN individuals (Supplementary Figure S3A–C, S4A,B, S5A; Figure 2A,B,D). CD4^+^ T cells negatively correlated with convalescence period (Figure 2C), except in a vaccinated COVID-19^+^ female (sample #15). Although the percentage of PD-1^+^ CD4^+^ T cells did not change in COVID-19^+^, the percentage of PD-1^+^ CD8^+^ T cells showed an increasing trend in COVID-19^+^ and negatively correlated with convalescence (Figure 2E–G, Supplementary Figures S6A, S7A). Frequency of antigen-specific CD154^+^CD69^+^CD8^+^ T cells did not differ between COVID-19^+^ and UN individuals but remained higher in early convalescence and decreased with increasing convalescence period (Figure 2H,I, Supplementary Figure S7A); HLA-DR^+^CD38^+^ hyperactivated CD8^+^ T cells also negatively correlated with convalescence (Figure 2J,K, Supplementary Figure S7A). Similarly, CD4^+^ T-cell subpopulations showed no baseline difference within the lungs of the study groups (Supplementary Figure S6A,E,F). Baseline evaluation of proliferating CD4^+^ and CD8^+^ T cells showed no notable difference between the study groups (Figure 2L,M, Supplementary Figures S6, S7B). The IFN-γ-secreting CD4^+^ T cells, along with those also expressing Ki-67^+^, trended higher in COVID-19^+^ than in controls (Figure 2N,P), whereas no difference in IFN-γ-secreting CD8^+^ T-cell subpopulations were observed at baseline (Figure 2O,Q). The maintenance of cell populations over the culture period was assessed, comparing UN or COVID-19^+^ tissues in culture to the respective starting tissues. Reduction in total CD45^+^, CD4^+^, and CD8^+^ T-cell frequencies was observed in both COVID-19^+^ and UN samples over the culture period (Supplementary Figure S8A–C); TNF-α^+^-producing CD45^+^ cells also decreased, but the change was not significant (Supplementary Figure S5B). Despite this, the functionality of these T cells (Ki-67^+^, IFN-γ^+^, CD69^+^, CD38^+^ etc.) did not decrease with culture in UN or COVID-19^+^ (Supplementary Figure S8D–O). Both CD4^+^ and CD8^+^ Ki-67^+^ cells, along with hyperactivated (HLA-DR^+^CD38^+^) CD4^+^ T cells, showed significant increase, and CD4^+^ PD-1^+^ showed an increasing trend in COVID-19^+^ with culture (Supplementary Figure S8D,E,H,J). Of the CD45^neg^ cells, endothelial cell frequencies did not change (Supplementary Figure S4C), whereas epithelial subpopulations differentially increased over time in both UN and COVID-19^+^ lung tissues (Supplementary Figure S3D).

Following exposure to the SARS-CoV-2 peptide pools, alterations in the cellular landscape and immune response were evaluated in the lung tissue cores. Epithelial cell populations were not altered significantly, but CD31^+^ endothelial cells responded to S peptide (in UN) and M peptide (in COVID-19^+^); a significant change was noted in COVID-19^+^ following M-peptide exposure compared to UN individuals (Supplementary Figure S3E, S4D). S-peptide exposure increased the overall percentage of CD45^+^ immune cells in COVID-19^+^ samples, when compared to control and S-peptide exposed UN samples (Figure 2R). Both CD3^+^CD4^+^ and CD3^+^CD8^+^ T cells showed an increasing trend in UN samples when compared to COVID-19^+^ with M- and S-peptide exposure (Figure 2S,T). Antigen-specific, hyperactive, and PD-1^+^ CD4^+^ and CD8^+^ T-cell frequencies were not significantly altered within or between groups following peptide stimulation (Supplementary Figure S6G–I, Supplementary Figure S7E–G), nor were CD45^+^TNF-α^+^ cells (Supplementary Figure S5C). The percentages of proliferating CD4^+^ (Ki-67^+^) T cells in COVID-19^+^ samples showed an increasing trend with N peptide when compared to control, and the proliferating CD4^+^IFN-γ^+^ T cells in COVID-19^+^ samples responded after M-peptide stimulation when compared to control (Figure 2U,Y); notable differences were not observed within each group in IFN-γ-secreting CD4^+^ T cells or proliferating or IFN-γ-secreting CD8^+^ T cells following peptide stimulation (Figure 2V–X,Z). Although exposure to SARS-CoV-2 peptides did not affect overall frequencies of IFN-γ^+^CD4^+^ T cells, patient-specific responses were noted. The largest changes were observed in UN subject #7, where a 2.8–3.8-fold increase in this cell population was found upon peptide stimulation, and COVID-19^+^ subjects #10 and #13, with a 1.7–1.8-fold and a 3.4-fold increase (M-peptide), respectively. M-peptide exposure showed a modest overall increase in IFN-γ^+^CD8^+^ T cells when COVID-19^+^ samples were compared to UN samples, and patient-specific responses to peptide stimulation were observed (Figure 2X). The largest changes were observed in UN subject #2, where a 13-fold increase was observed following N-peptide stimulation, and COVID-19^+^ subjects #10 and #13, with a 2-fold (S peptide) and 3.2-fold (M peptide) increase, respectively.

### Memory T-cell response to SARS-CoV-2 peptides within lung tissues of convalescent individuals

**FIGURE 3:**
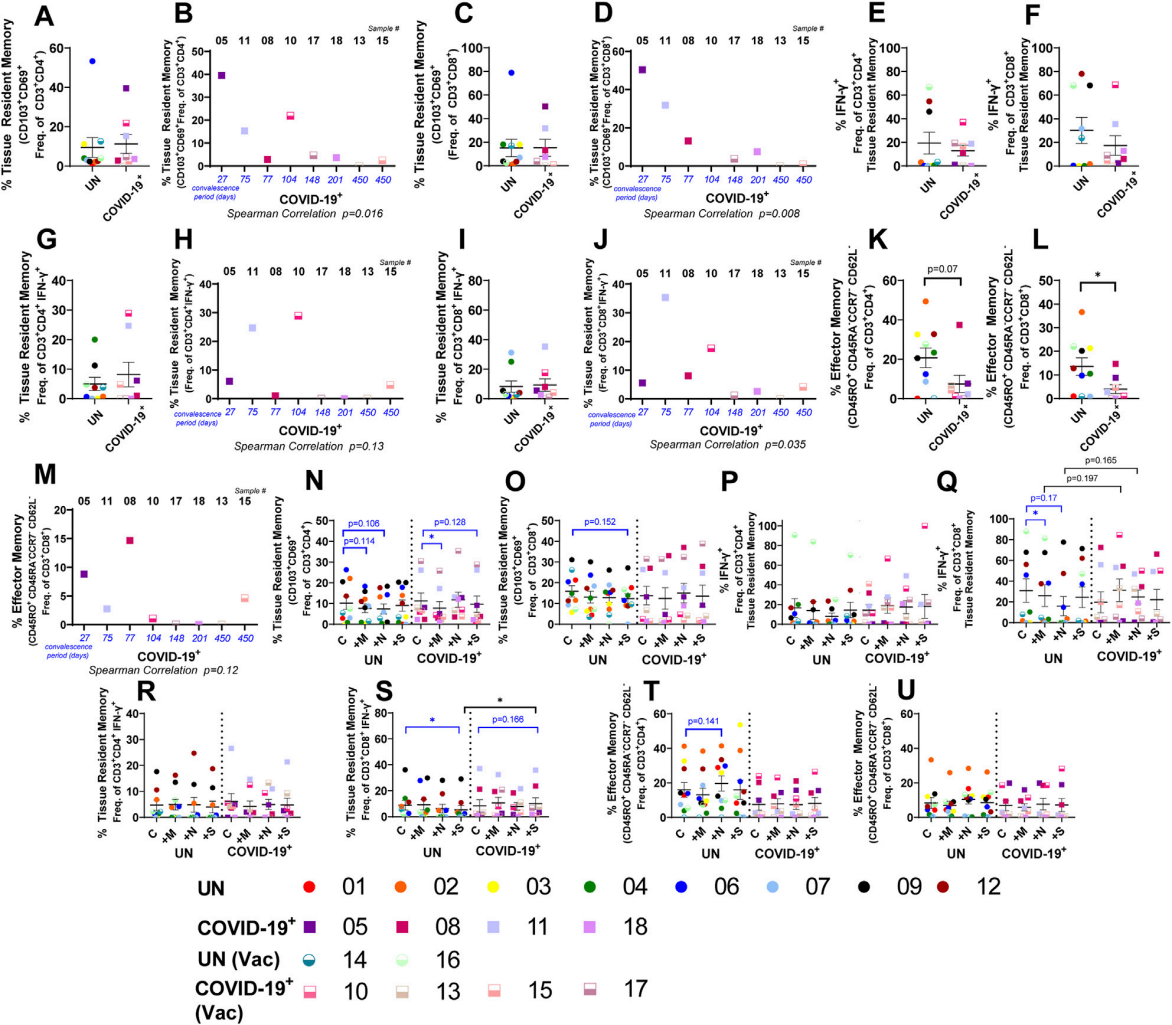
Memory T-cell response to SARS-CoV-2 peptides within the lung. **(A**, **C**, **E–G**, **I**, **K**, and **L)** Baseline differences in memory T cells within lung tissue from uninfected (UN) individuals and individuals recovering from COVID-19 (COVID-19^+^). **(B**, **D**, **H**, **J**, and **M)** Correlation between the convalescence period for COVID-19^+^ samples and the percentage of CD4^+^ tissue-resident memory (TRM) T cells **(B)**, CD8^+^ TRM T cells **(D)**, TRM IFN-γ^+^ CD4^+^ T cells **(H)**, TRM IFN-γ^+^ CD8^+^ T cells **(J)**, and effector-memory CD8^+^ T cells **(M)**. **(N–U)** Impact of SARS-CoV-2 peptide exposure on memory T-cell populations in UN and COVID-19^+^ lung tissues (n = 10 UN and n = 8 COVID-19^+^ [mean (center line) ± SEM]). Statistics shown in blue are comparisons between control and peptide-exposed samples within each group (UN and COVID-19^+^). Statistics shown in black are the change in the response between UN and COVID-19^+^ for each peptide when compared to the corresponding control.

We next assessed T-cell memory subsets within the lung tissues of UN and COVID-19^+^ individuals, as they play a vital role in viral clearance during re-infection and recent studies identified functional memory T cells within the peripheral blood of COVID-19^+^ patients (Lipsitch et al., 2020; Ni et al., 2020; Peng et al., 2020; Rydyznski Moderbacher et al., 2020; Jung et al., 2021; Rodda et al., 2021; Tavukcuoglu et al., 2021). At baseline, CD4^+^ and CD8^+^ naïve, stem cell-like memory, and central-memory T-cell subsets were not significantly different when comparing UN and COVID-19^+^ tissues (Supplementary Figure S9A–I). TRM and effector memory (EM) cells were the most prevalent subsets within the lung tissue (Figures 3A,C,K,L, and Supplementary Figure S9B). While the baseline levels of TRM T cells and TRM subsets did not differ between UN and COVID-19^+^ (Figure 3A, C, E–G, I), the percentages of both CD4^+^ and CD8^+^ TRM T cells negatively correlated with the convalescence period (Figure 3B,D). Furthermore, the TRM cells within the IFN-γ^+^CD8^+^ population negatively correlated with convalescence, and a similar trend was observed with TRM cells within the IFN-γ^+^CD4^+^ population (Figure 3H,J). Importantly, baseline reduction in both CD4^+^ and CD8^+^ EM cells were observed in COVID-19^+^ compared to UN individuals (Figure 3K,L), and CD8^+^ EM cells tended to negatively correlate with convalescence (Figure 3M). CD4^+^ and CD8^+^ memory T-cell populations did not change with culture (Supplementary Figure S9A, P-AC). Following *ex vivo* culture with peptide pools, the total CD4^+^ TRM cell frequencies decreased significantly in response to M peptide in COVID-19^+^ tissues and showed a trending decrease in response to S peptide; a modest reduction was also noted in UN tissues (Figure 3N), but overall CD8^+^ TRM cell frequencies remained unchanged following peptide stimulation (Figure 3O). We determined the percentage of IFN-γ^+^ cells within the CD4^+^ and CD8^+^ TRM subsets and the percentage of TRM cells within the overall IFN-γ^+^ CD4^+^ and CD8^+^ subsets. Although statistical significance was not observed when comparing UN and COVID-19^+^ samples, patient-specific differences were noted for peptide responses in COVID-19^+^ (Figure 3P–S). Within COVID-19^+^, robust increases in the percentages of IFN-γ^+^CD4^+^TRM and IFN-γ^+^CD8^+^TRM in response to S peptide (8- and 50-fold, respectively, compared to 0 in controls) were noted only in subject #10 (female COVID-19^+^ with the shortest convalescence vaccinated at 27 days before tissue resection) (Figure 3P,Q). Additional peptide responses were noted with this individual, including 5-fold (IFN-γ^+^CD4^+^TRM) and 84.6-fold (IFN-γ^+^CD8^+^TRM) increases in response to M peptide as well as 2.25-fold (IFN-γ^+^CD4^+^TRM) and 50-fold (IFN-γ^+^CD8^+^TRM) increases in response to N peptide, suggesting an overall increase in recall responses in this individual soon after vaccination (Figure 3P,Q). Interestingly, although the percentage of TRM cells within CD4^+^CD3^+^IFN-γ^+^ and CD8^+^CD3^+^IFN-γ^+^ did not show a robust response with S-peptide in this individual, the percentage of TRM within CD8^+^CD3^+^IFN-γ^+^ increased in response to M- and N-peptides (7.81- and 7.25-fold, respectively, compared to 0 in controls) were also noted in subject #10 (Figure 3R,S). Out of the two-dose-vaccinated COVID-19^+^ subjects, subject #13 with the longest convalescence period, showed 7.99- and 4.98-fold increases in the percentages of IFN-γ^+^CD4^+^TRM and IFN-γ^+^ CD8^+^TRM, respectively, in response to M peptide and 3.05- and 4.83-fold increases, respectively, in response to N peptide (Figure 3P,Q). Interestingly, the percentage of TRM cells within CD4^+^CD3^+^IFN-γ^+^ and CD8^+^CD3^+^IFN-γ^+^ of this individual did not show a robust response with M peptide, but 2.2–2.28-fold changes were observed in response to N peptide, and modest 1.22–1.26-fold differences were found in these populations in response to S peptide. However, in two-dose-vaccinated and boosted female COVID-19^+^ subject #17, the percentage of TRM within CD8^+^CD3^+^IFN-γ^+^ showed 20.83- and 6.67-fold increase following M-peptide and N-peptide exposures, respectively (Figure 3S). Additionally, subject #15 (two-dose-vaccinated and boosted male subject with the longest convalescence), did not demonstrate a robust increase in any of the TRM cell populations in response to M, N, or S peptides (Figure 3P–S). On the contrary, in subject #18, an unvaccinated convalescent male subject, the percentages of IFN-γ^+^CD4^+^TRM and IFN-γ^+^ CD8^+^TRM increased 20.6-fold and 18.95-fold, respectively, in response to N peptide only (Figure 3P–Q).

The negative correlation of TRM cells within the IFN-γ^+^CD8^+^CD3^+^ population with convalescence period (Figure 3J) indicates that the induction of functional lung TRM T cells occurs early following infection. The response of IFN-γ^+^CD8^+^CD3^+^TRM to one peptide highly correlated with the response to peptide stimulation with the other two peptides in COVID-19^+^ (Supplementary Table S3). Although TRM responses to peptides occurred mostly in female COVID-19^+^, statistical correlation with sex was not observed (Supplementary Table S3). The stem cell-like memory, effector memory, central memory, and naïve T-cell frequencies were not significantly altered in response to peptide exposure in COVID-19^+^ or UN tissues (Figure 3T,U, Supplementary Figure S9J–O).

### Humoral immune response to SARS-CoV-2 peptides within lung tissues of convalescent individuals

**FIGURE 4:**
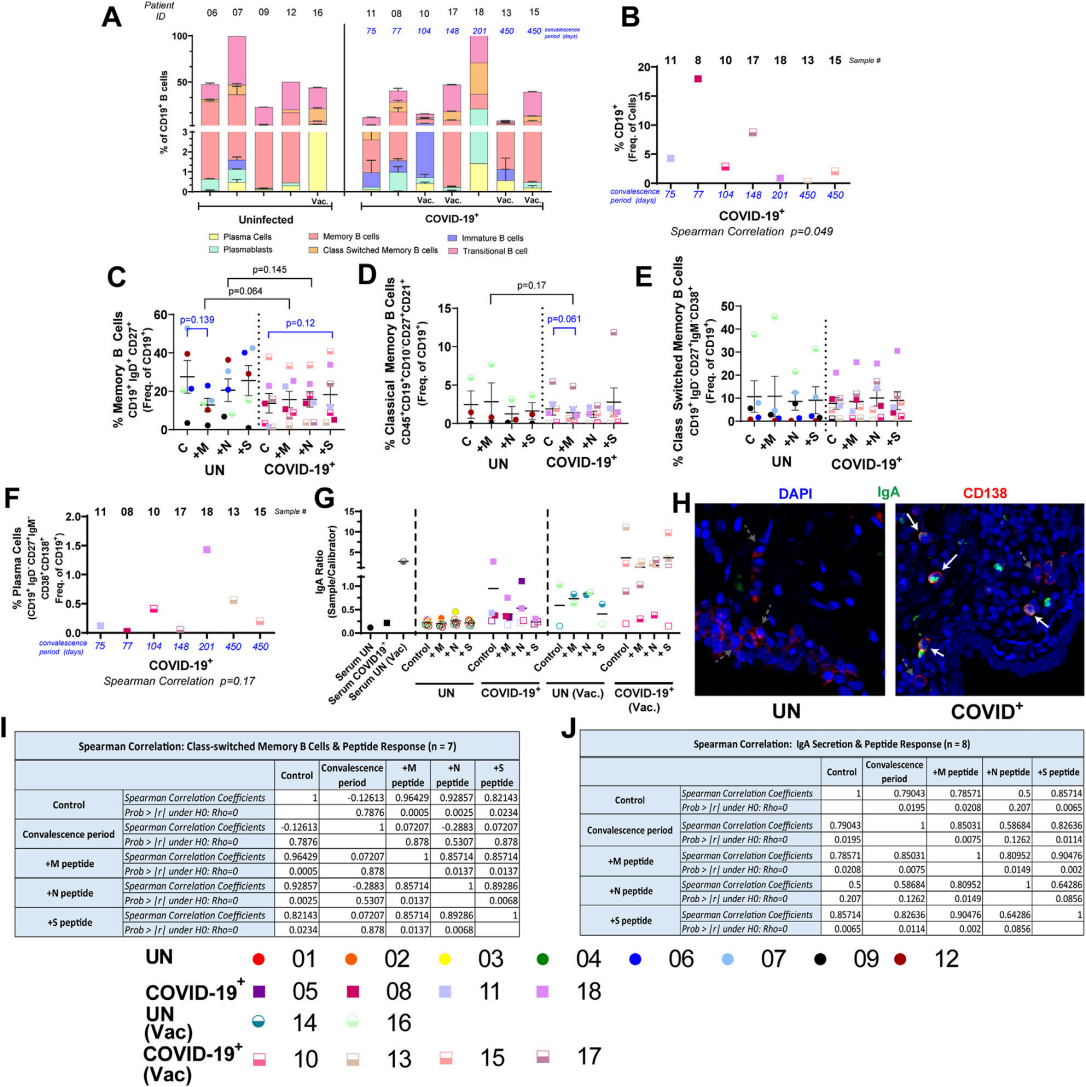
Local B-cell response to SARS-CoV-2 peptides within the lung tissues. **(A)** Baseline differences in B-cell populations within lung tissue from uninfected (UN) individuals and individuals recovering from COVID-19 (COVID-19^+^). **(B)** Correlation between the convalescence period for COVID-19^+^ samples and the percentage of CD19^+^ B cells. **(C–E)** Impact of SARS-CoV-2 peptide exposure on B-cell populations in UN and COVID-19^+^ lung tissues. **(F)** Correlation between the convalescence period for COVID-19^+^ samples and the percentage of plasma cells. **(G)** Quantification of circulating SARS-CoV-2-specific IgA; opaque symbols indicate positive samples (measurements over threshold). **(H)**. Photomicrographs showing CD138^+^ plasma cells (red) secreting IgA (green) in UN (left) and COVID-19^+^ samples (right) at day 0 (starting tissue). White arrows pointing to CD138^+^ plasma cells secreting IgA, gray dashed arrows pointing to CD138^+^ plasma cells without IgA secretion. **(I** and **J)**. Correlation table showing correlation among the convalescence period, peptide response, and class-switched memory B populations **(I)** and the convalescence period, peptide response, and IgA secretion **(J)** (n = 5–10 UN and n = 6–8 COVID-19^+^ [mean (center line) ± SEM]). Statistics shown in blue are comparisons between control and peptide-stimulated samples within each group (UN and COVID-19^+^). Statistics shown in black are the change in response between UN and COVID-19^+^ for each peptide when compared to the corresponding controls.

Humoral immune response to SARS-CoV-2 infection is essential for protection (Cagigi et al., 2021; Lapuente et al., 2021; Ruiz et al., 2022); localized responses in the lung tissue during SARS-CoV-2 convalescence is largely unknown. We evaluated B-cell subsets in a subset of the UN and COVID-19^+^ lung tissues. Baseline differences in B-cell subsets, memory B-cell subsets, plasma cells, or plasmablasts were not observed in the study groups (Figure 4A, Supplementary Figure S10A–M); however, modest reduction in the percentage of transitional B cells and % immature transitional B cells in the COVID-19^+^ group was observed (Supplementary Figure S10I,J). Significant changes due to culture were not observed in B-cell populations, except for activated memory B and classical memory B cells, in which an increasing trend was noted in COVID-19^+^, and atypical memory B cells, in which a significant increase was observed in COVID-19^+^ (Supplementary Figure S11). Although the overall percentage of CD19^+^ B cells negatively correlated with convalescence period (Figure 4B), following *ex vivo* culture and peptide exposure, the frequency of CD19^+^ B cells did not change in either group compared to relevant controls (Supplementary Figure S10O). The percentage of memory B cells in M- or N-peptide-exposed COVID-19^+^ lung tissues showed an increasing trend when compared to UN tissues (Figure 4C). A decreasing trend was noted for the percentage of classical memory B cells in M-peptide-exposed COVID-19^+^ lung tissues compared to control and M-peptide exposure in UN tissues (Figure 4D). The percentages of immature B cells, activated memory B cells, and transitional B cells were not altered following peptide exposure (Supplementary Figure S10P,R,T). The percentage of class-switched memory B cells also did not change with peptide exposure, nor were they correlated with convalescence period (Figure 4E, Supplementary Figure S10N); the class-switched memory B-cell frequency was the highest in a COVID-19^+^ male who was not vaccinated and at 201 days of convalescence when the tissue sample was obtained. A decreasing trend in the percentage of naïve B cells in COVID-19^+^ lung tissues was noted following peptide exposure when compared to controls (Supplementary Figure S10Q), and a decreasing trend in the percentage of atypical memory B cells was observed when M- or S-peptide exposed samples were compared between COVID-19^+^ and UN samples (Supplementary Figure S10S). The percentage of immature transitional B cells increased with M-peptide exposure in COVID-19^+^ compared to UN samples, and there was an increasing trend following S-peptide exposure in COVID-19^+^ compared to control (Supplementary Figure S10U). Although plasmablasts were not significantly different at baseline or in response to the peptide exposure, a decreasing trend in plasma cells with M-peptide exposure was noted in COVID-19^+^ samples when compared to UN samples (Supplementary Figure S10L,V,W). Additionally, the frequencies of plasma cells showed a trend in positive correlation with convalescence period (Figure 4F); the highest plasma cell frequency was in the same COVID-19^+^ male at 201 days of convalescence (#18) who had the highest frequency of class-switched memory B cells (Figure 4F). Evaluation of humoral responses in the lung tissue identified higher baseline IgA levels in 3/4 of vaccinated COVID-19^+^ (subjects #13, #15, and #17) compared to the unvaccinated COVID-19^+^ (subjects #11, #5, #8, and #18, see Supplementary Table S2); both groups had higher IgA levels than UN samples (Figure 4G). S-peptide exposure increased IgA levels in vaccinated #15, and N-peptide stimulation increased IgA levels in subject #17 when compared to baseline (Figure 4G). Of the COVID-19^+^ with high baseline IgA, unvaccinated subject #18 showed the lowest IgA following peptide stimulation (Figure 4G). Baseline IgA was lower in COVID-19^+^ subject #10 (one-dose vaccine and lowest convalescence), but both M- and N-peptide stimulation increased IgA levels (Figure 4G). IgA was not detectable at baseline in COVID-19^+^ subject #5 (infected once and unvaccinated), but both M- and N-peptide stimulation increased IgA levels substantially (Figure 4G). Overall, the IgA response was more dominant than the IgG response in supernatants of COVID-19^+^ tissue models (Supplementary Figure S10X). We then used confocal microscopy to identify CD138^+^ plasma cells secreting IgA in the lungs of COVID-19^+^ compared to those of UN individuals. Consistent with SARS-CoV-2-specific IgA levels noted in unvaccinated and vaccinated COVID-19^+^, we detected more CD138^+^IgA^+^ cells in the lungs of COVID-19^+^ at baseline and in response to SARS-CoV-2-specific peptides [Figure 4H, Supplementary Figure S12, and Supplementary Video S1 (https://figshare.com/s/4791622c8dfcd89f0aea)]. Importantly, in culture supernatants of lung tissues from COVID-19^+^, IgA levels positively correlated with convalescence without peptide exposure and with M- or S-peptide exposures (Figure 4J). Additionally, IgA response to one peptide positively correlated with the response to the other peptides and convalescence (Figure 4J). When class-switched memory B-cell responses were assessed in COVID-19^+^, response to one peptide also positively correlated with the response to the other peptides (Figure 4I). No correlation between the percentage of class-switched memory B cells and IgA production was observed (Supplementary Table S4).

## Discussion

T-cell dynamics during both the acute and convalescent phases following SARS-CoV-2 infection determine the specific T-cell and humoral responses optimized for host protection. Specifically, the role of SARS-CoV-2-specific effector T cells on viral clearance and their dynamics following COVID-19 requires further study; however, modeling local immune responses against SARS-CoV-2 has posed challenges. In this study, we utilized a novel 3D perfused human lung-tissue model that maintains cellular heterogeneity, viability, and extracellular matrix components over an extended culture period. This *ex vivo* culture model has the advantage of maintaining the native tissue architecture of the human lung to allow for tissue-level alterations to be assessed in a representative microenvironment, better replicating tissue structure and cell–cell interactions than lung organoids, which are often limited due to the absences of immune and stromal cells, as well as the native tissue ECM (Salahudeen et al., 2020; Lee et al., 2023). This 3D tissue-culture model allowed for *ex vivo* studies on lung-tissue specimens from both uninfected controls and COVID-19-convalescent study subjects undergoing lobectomy or wedge resection for removal of lung nodules/masses. Remnant adjacent uninvolved lung specimens removed during such resections were used in our *ex vivo* culture platform, as such tissue samples would not be otherwise available from healthy donors. The tissue specimens utilized were obtained from the sampled region furthest away from the lung masses. In this unique model, local immune responses to SARS-CoV-2 peptide pools that represent membrane, nucleocapsid, and spike proteins of SARS-CoV-2 were evaluated. Evidence for pre-existing T-cell immunity and SARS-CoV-2 peptide-specific local T- and B-lymphocyte memory responses in lung tissues from COVID-19^+^ and UN individuals was found within human lung explant models.

Frequencies of PD-1^+^ CD8^+^ T cells showed increasing trends in our COVID-19^+^ lung tissue at baseline and their decrease with convalescence are consistent with the reported increase in circulating PD-1^+^ cells in COVID-19^+^ (Gong et al., 2020; Boppana et al., 2021; Kusnadi et al., 2021; Rha et al., 2021). Additionally, as observed in SARS-CoV-2-infected individuals (Mathew et al., 2020; Thevarajan et al., 2020), the presence of hyperactivated CD4^+^ and CD8^+^ T cells and the decrease in the CD8^+^ subset with convalescence are recapitulated in our tissue model. Importantly, through the use of *ex vivo* lung models, we report M-peptide-responding functional IFN-γ-secreting and proliferating CD4^+^ T cells in recovering COVID-19^+^. Although not significant, pre-existing T-cell immunity in UN lungs was observed with M-peptide responses in proliferating CD8^+^ T cells, and a trending increase in difference of CD8^+^ T-cell response to M-peptide in UN samples was observed, as reported earlier in circulation (Parveen et al., 2019; Grifoni et al., 2020a; Grifoni et al., 2020b; Kared et al., 2020; Le Bert et al., 2020; Meckiff et al., 2020; Ong et al., 2020; Rydyznski Moderbacher et al., 2020; Sattler et al., 2020; Files et al., 2021; Nelde et al., 2021; Schulien et al., 2021).

Previous studies on SARS-CoV-recovered and COVID-19-recovered and/or vaccinated individuals have identified persistent memory T cells, suggesting that the induction of TRM cells may provide long-term protection against the current pandemic (Channappanavar et al., 2014; Grau-Expósito et al., 2021; Poon et al., 2021; Pieren et al., 2023). Consistent with this, CD8^+^ EM T cells and those of the TRM phenotype have recently been identified in bronchoalveolar lavage fluid and lung tissues of individuals with COVID-19 (Liao et al., 2020; Grau-Expósito et al., 2021). TRM cells are known to provide protection against pathogen challenge in many tissues, including the lungs (Channappanavar et al., 2014; Wu et al., 2014; Liao et al., 2020; Grau-Expósito et al., 2021). We report here the presence and long-term maintenance of SARS-CoV-2-specific TRM and EM CD8^+^ T cells in COVID-19^+^ lungs. In our studies, the average percentages of CD4^+^TRM, CD8^+^TRM, and IFN-γ^+^CD8^+^TRM negatively correlated with convalescence. This is not entirely surprising as the TRM population is likely in part driven by SARS-CoV-2-specific TRM cells, which are expected to decrease with the convalescence period, as has been observed in murine models of other infections (Wu et al., 2014). Additionally, studies linking a high proportion of lung TRM T cells with lung pathologies (IPF and COPD) and poor lung function have been described (Cheon et al., 2021; Serezani et al., 2022; Carbone, 2023); therefore, there may be a biological benefit to reducing this cell population following viral insult to maintain lung homeostasis. Additionally, Davis-Porada et al. (2024) have shown that S-reactive CD4^+^ TRM decrease with age; therefore, we cannot discount that patient age could be a factor in our observations. Although all the vaccinated COVID-19^+^ demonstrated an overall increase in response to SARS-CoV-2 peptides, the responses were mostly associated with M and N peptides; a substantial increase in S-peptide responses in TRM cells was noted only for the vaccinated COVID-19^+^ with the shortest convalescence. The increase in S-peptide-responding TRM cells is consistent with vaccine-mediated induction of TRM cells as a strategy for potential long-term protection (Channappanavar et al., 2014; Poon et al., 2021; Roukens et al., 2022; Tang et al., 2022; Pieren et al., 2023; Rotrosen and Kupper, 2023). The TRM response that was more prominent in early convalescence is consistent with the induction of functional TRMs in the early phase after infection (Carbone, 2023). The TRM response to peptide was higher in the vaccinated and boosted convalescent individual than in the vaccinated but not boosted individual, suggesting that boosting the immune response with additional vaccine doses may reinvigorate and enhance the recall response. Additionally, vaccine-induced TRM cells localized to lung tissue of COVID-19^+^, suggesting potential beneficial effects in the respiratory tract. Furthermore, the percentage of TRM cells in IFN-γ^+^ T cells was higher in COVID-19^+^ at baseline and that of IFN-γ^+^ TRM cells was higher in response to peptide pools in COVID-19^+^ than in UN individuals. In this context, high frequencies of spike protein-specific CD4^+^T cell responses have been reported in blood samples of COVID-19 convalescent (Grifoni et al., 2020a; Grifoni et al., 2020b; Le Bert et al., 2020; Ni et al., 2020; Sekine et al., 2020). CD4^+^T cells are necessary for the formation of protective CD8^+^ TRM cells during influenza infection; IFN-γ is an essential signal for this process (Oja et al., 2018). Here, both IFN-γ^+^ CD4^+^ and CD8^+^ TRM cells were responding to both M and N peptides in addition to S-peptide pools. These data suggest that the nucleocapsid and/or structural viral proteins in vaccines may broaden the profile of memory T cells, commonly observed during control of natural infection.

Additionally, localized B-cell responses to SARS-CoV-2 peptide pools against spike protein in COVID-19^+^ lung models are reported, with an increasing trend in the percentage of M-peptide responding memory B cells in COVID-19^+^ compared to UN individuals, and the S-peptide response trended higher within COVID-19^+^ than in controls; it remains to be determined whether they are tetramer^+^ antigen-reactive B cells. Importantly, we noted increased IgA levels and IgA^+^ plasma cells in the lungs of COVID-19^+^ compared to UN individuals, both at baseline and in response to SARS-CoV-2 peptides; vaccinated individuals had a higher IgA response at baseline and post-S-peptide exposure. Class-switched memory B cells are key components of the reactive humoral immunity, which ensures a fast and massive secretion of high-affinity antigen-specific antibodies upon antigenic challenge (Akkaya et al., 2020; Goel et al., 2021; Çölkesen et al., 2022). In humans, IgA class-switched memory B cells and IgA antibodies are abundant in the blood (Akkaya et al., 2020). Consistent with this, we show a positive correlation between IgA presence in lung tissue and the convalescence period in COVID-19^+^ subjects. Additionally, we show that the convalescence period positively correlates with IgA production in the lung tissue with localized responses to N or S peptides of SARS-CoV-2. Thus, in vaccinated subject #10, due to the early convalescence period, IgA class switching may not have occurred; this is consistent with this individual’s reduced percentage of class-switched memory B cells and IgA levels in the lungs. As secretory IgA plays a crucial role in protecting mucosal surfaces against respiratory viruses and other pathogens, vaccine approaches via nebulization or intranasal delivery may enhance IgA levels in the upper airways and provide long-term protection against COVID-19.

## Conclusions

Together, our data demonstrate the utility of the 3D lung explant model in evaluating local immune response dynamics following COVID-19 within human lung tissues. These human lung explant models show evidence of pre-existing T-cell immunity and SARS-CoV-2 peptide-specific local T- and B-cell memory responses in COVID-19-convalescent and previously uninfected individuals. This includes the long-term maintenance of TRM and EM CD8^+^ T cells in COVID-19-convalescent lungs and increased IgA levels and IgA^+^ plasma cells in the lungs of COVID-19 convalescents, both at baseline and in response to SARS-CoV-2 peptides. Although this model has the benefit of maintaining the native tissue architecture, it does not enable the evaluation of immune cell recruitment in response to peptide stimulation. Additionally, this study was not able to capture SARS-CoV-2-specific T- or B-cell populations; therefore, antigen specificity was not evaluated.

## Methods

### Sex as a biological variable

Tissue specimens from both male and female individuals were used in this study, and sex was considered a biological variable.

### Clinical sample collection

De-identified, remnant surgical specimens were obtained from lobectomy and wedge resection surgeries performed at the University of Alabama at Birmingham (UAB) through the UAB Tissue Biorepository. For peptide exposure studies, 10 tissue specimens were obtained from patients with no history of SARS-CoV-2 infection, and eight tissue specimens were obtained from patients who had previously tested positive for SARS-CoV-2 and cleared the infection. Specimens were obtained from patients undergoing tissue resections due to lung nodules (95%) or transplant (5%). Samples were collected from patients with no known chronic lung diseases, and the tissue used for this study was considered uninvolved, adjacent lung tissue, obtained from a region away from the lung nodule that appeared the healthiest upon gross examination by the pathology assistant. This study was approved by the University of Alabama at Birmingham Institutional Review Board (IRB-300003092 and IRB-300003384) and conducted following approved guidelines and regulations. All samples for peptide exposure studies were collected between August 2020 and July 2022 in the early stage of the pandemic. Thus, samples from infected controls could be collected. In the early phase of the pandemic, vaccines and boosters were also efficiently tracked. Patient demographics are described in Supplementary Table S1, and sample collection statistics over time are shown in Supplementary Figure S13.

### Sample processing and *ex vivo* perfusion culture

Tissue cores with a diameter of 5 mm were generated from remnant surgical specimens using a tissue coring press (Alabama Research and Development, United States). One tissue core was placed into the central chamber of a polydimethylsiloxane (PDMS, Krayden, United States) bioreactor (measuring 8 × 6 × 10 mm), containing a mixture of ECM [90% collagen type 1 (Advanced Biomatrix, United States) + 10% growth factor-reduced Matrigel (Corning, United States)] components for structural support, as previously described (Goliwas et al., 2017). The tissue/ECM volume was then penetrated with five 400-micron Teflon-coated stainless steel wires to generate through-channels for tissue perfusion. Following ECM polymerization, the wires were removed, and the through-channels were filled with tissue culture media (1:1 mixture of X-Vivo15 and bronchial epithelial growth media [Lonza, United States) with antibiotics (MP Biomedicals, United States)]. The bioreactor was then connected to a perfusion system, which contained a media reservoir, peroxide-cured silicon tubing (Cole Parmer, United States), a collection reservoir, and a peristaltic pump (ESI, United States). Tissue culture media were perfused through the tissue volume for 5–14 days (37°C, 5% CO_2_), with media changed every 3 days. At the end of each experiment, the tissue was split, with a portion of each tissue fixed for histological processing and a portion undergoing collagenase B (Roche, Switzerland) digestion for flow cytometry analysis.

### SARS-CoV-2 peptide exposures

For *in vitro* peptide exposures, on day 5 of culture, conditioned media were collected from the collection reservoir, and according to the manufacturer’s instructions, 0.6 nM SARS-CoV-2 peptides (PepTivator peptide pools: M, N, and S, Miltenyi Biotec, United States) or vehicle control (cell culture grade water) was added to the tissue chamber of the bioreactor. Following a 4-hour static incubation, tissue culture media containing brefeldin A and monensin (BD, Germany) were perfused throughout the tissue chamber for an additional 14 h (unless otherwise noted).

### Multiparametric flow cytometry

The following antibodies were used for multiparametric flow cytometry for T-cell analysis: Anti-CD3-Alexa Fluor 700 (Clone: UCHT1) and anti-CD69-BUV563 (Clone: FN50) from BD Biosciences (Germany); anti-HLA-DR-APC (Clone: LN3), anti-CD4-FITC (Clone: RPA-T4), anti-CD3-PE-Cy7 (Clone: UCHT1), anti-CD8-APC (Clone: OKT8), and anti-Interferon-γ-Alexa Fluor700 (Clone: B27) from eBioscience (Thermo Fisher, Germany); anti-Ki-67-Dylight350 (Clone: 1297A) from Novus (United States); anti-CD45-APC-Cy7 (Clone: 2D1), anti-CCR7-Pacific Blue (Clone: G043H7), anti-CD45RA-BV510 (Clone: HI100), anti-CD45RO-PerCy-Cy5.5 (Clone: UCHL1), anti-CD62L-BV650 (Clone: DREG-56), anti-CD103-PE (Clone: Ber-ACT8), anti-CD8-BV510 (Clone: SK1), anti-CD38-PE-Cy7 (Clone: HB-7), anti-CD154-PE/Dazzle (Clone: 24–31), anti-PD-1-BV605 (Clone: NAT105), and anti-TNF-αBV605 (Clone: MAb11) from BioLegend (United States).

The following antibodies were used for multiparametric flow cytometry for analysis of resident immune and structural cells: Anti-CD64-PerCp-eFluor710 (Clone: 10.1) and anti-HLA-DR-FITC (Clone: LN3) from eBioscience (Thermo Fisher, Germany); anti-CD16-PE (Clone: 3G8), anti-CD45-Pacific Blue (Clone: HI30), anti-CD66b-PerCP-Cy5.5 (Clone: G10F5), anti-EpCAM(CD326)-Alexa Fluor 594 (Clone: 9C4), and anti-CD31-Alexa Fluor 700 (Clone: WM59) from BioLegend (United States); anti-CD11b-APC-Cy7 (Clone: ICRF44) from BD Biosciences (Germany); anti-PanCytokerain-APC (Clone: C-11) and anti-Ki-67-Dylight350 (Clone: 1297A) from Novus Biologicals (United States).

The following antibodies were used for multiparametric flow cytometry for B-cell analysis: Anti-CD19-eFluor450 (Clone:HIB19) from eBioscience (Thermo Fisher, Germany); anti-CD10-BV650 (Clone:HI10A) from BD Biosciences (Germany); anti-CD45-BV605 (Clone: 2D1), anti-CD21-PerCP-Cy5 (Clone:Bu32), anti-CD24-APC-Cy7 (Clone:ML5), anti-CD27-APC (Clone: M-T271), anti-CD38-PE-Cy7 (Clone: HB-7), anti-CD138-BV510 (Clone:MI15), anti-IgD-FITC (Clone:IA6-2), and anti-IgM-PE (Clone:MHM-88) from BioLegend (United States).

The Foxp3/Transcription Factor Staining Buffer Set (Thermo Fisher, Germany) and the Cytofix/Cytoperm Fixation/Permeabilization Kit (BD, Germany) were used, according to the manufacturer’s protocol, to stain for intracellular molecules (intranuclear and cytoplasmic molecules, respectively). Fluorescence minus one (FMO) controls were used as shown in Supplementary Figure S14. Analyses were performed using the FACSymphony A3 Cell Analyzer with FACSDiva software version 8.0.1 (BD Biosciences, Germany). Data were analyzed using FlowJo 10.7.1 (Treestar, United States).

### Analysis of IgA and IgG

Analysis of secreted Sars-CoV-2-specific IgA and IgG was performed using conditioned media and serum, along with ELISA kits (EUROIMMUN). Conditioned media were concentrated using the Amicon Ultra-4 100 K Centrifugal Filter Units (Millipore Sigma), prior to analysis. Samples were spun at 4,000 × g for 15 min using a swinging bucket centrifuge, according to the manufacturer’s recommendation. ELISAs were performed following the manufacturer’s recommendations (1:101 dilution of serum samples and 1:5 dilution of conditioned media samples), and a threshold was set to determine the positive level of immunoglobulin based on the average level of immunoglobulin in control medium samples.

### Histologic processing and analysis

Following *ex vivo* culture, a portion of each cultured tissue was fixed with neutral buffered formalin, processed to paraffin, and histological (FFPE) sections were prepared, as previously described (Goliwas et al., 2016). Five-micron sections were stained with hematoxylin and eosin (H&E) to evaluate tissue morphology and cell density (number of cells per cross-sectional area), as described previously (Goliwas et al., 2021).

### Immunofluorescence staining and analysis

Immunofluorescence staining was performed on FFPE sections to detect plasma cells secreting IgA. Anti-CD138 (Syndecan-1, 1:50, clone aa18-218, LSBio) was detected with anti-mouse Alexa Fluor 647 (1:500 Life Technologies), and IgA (1:100, clone Mc24-2E11, LSBio) was detected with anti-rabbit Alexa Fluor-594 (1:500, Life Technologies), following antigen retrieval (10 mM citrate buffer, pH 6). 4′,6-Diamidino-2-phenylindole (DAPI) (1:1000) was used as a nuclear counterstain. Sections were imaged using a Nikon A1R-HD25 confocal microscope with a Plan Apo λ ×10 objective (na 0.5 wd 4,000).

### Matrix proteomics

For matrix protein enrichment and extraction, tissue samples were prepared as described previously (Naba et al., 2015). In brief, tissues were processed using the Millipore Compartment Protein Extraction Kit, with some modifications of the described methodology (Naba et al., 2015), and all fractions were stored at −80° overnight. The ECM fraction was then reconstituted in 8M urea and deglycosylated. The urea-insoluble fraction was collected through centrifugation, reconstituted in 1× LDS sample buffer, and sonicated for 20 min in an ultrasonic water bath. Both urea-soluble and insoluble fractions were quantified via the EZQ protein assay, and an equal amount per sample was loaded onto 10% Bis–Tris gels; gels were stained overnight with Colloidal Coomassie. Each sample was then digested into three fractions with trypsin overnight, and high-resolution LC-ESI-MS/MS analysis was completed. Data were searched against the human subset of the Uniref100 database with carbamidomethylation, oxidation, and hydroxyproline.

### Measuring lactate dehydrogenase

LDH was measured in conditioned media using the Invitrogen CyQUANT LDH Cytotoxicity Assay (Thermo Fisher, Germany), following the manufacturer’s instruction.

### Statistical analysis

The measured flow cytometry data were summarized by presenting descriptive statistics, such as mean with standard error of the mean (SEM), in the uninfected and convalescent groups. Changes between control and peptide-treated samples were computed separately. Two sample t-tests and Wilcoxon rank-sum tests were performed to determine whether means of the changes were different between uninfected and convalescent groups. Mean and SD of the outcome measured were estimated using control and peptide-treated samples within uninfected and convalescent groups, respectively. To evaluate differences in the outcome between control and each of the peptide-treated samples within each of the groups, paired t-test and Wilcoxon signed-rank tests were used. Spearman’s rank correlation between Day 0 and the convalescence period, between IgA, convalescence period, and class-switched memory cell percentage, and between sex, peptide response, and IFN-γ^+^ CD8^+^ TRM cells were evaluated for COVID-19^+^ samples. Statistical analyses were performed using SAS 9.4 (SAS Institute, United States). Statistical significance was determined at P-value <0.05.

## Data Availability

The raw data supporting the conclusions of this article will be made available by the authors, without undue reservation.
