# Supplementary material for: Lung-resident SARS-CoV-2 peptide-specific immune responses in perfused 3D human lung explant models
Source: Front Bioeng Biotechnol. 2025 Jul 8;13:1587080. doi: 10.3389/fbioe.2025.1587080 (PMC12279861; doi:10.3389/fbioe.2025.1587080)
Supplement: Supplementary file 1 [file DataSheet1.pdf]

Supplementary Figure 1

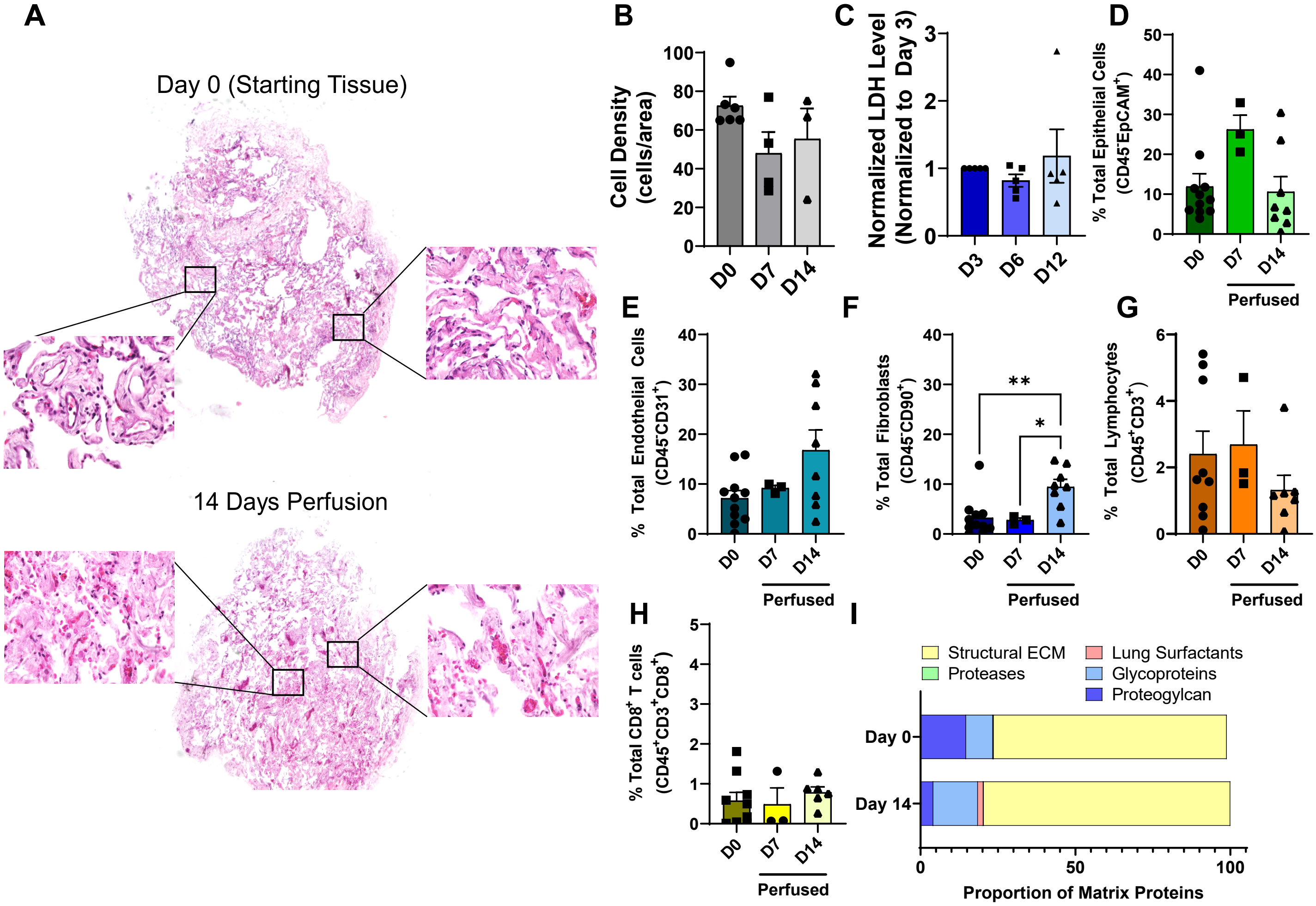

Supplementary Figure 1: *Ex Vivo* Maintenance of Lung Tissue over 14 Days Culture. **A**. Histologic architecture of human lung tissue is maintained following 14 days *ex vivo* culture. **B**. Lactate dehydrogenase (LDH) levels are stable during the culture period, indicating no increase in cell death during culture. **C-H**. Frequency of cell populations within the lung, comparing starting tissue to tissues cultured *ex vivo* for 7 or 14 days. **I.** The proportion of extracellular matrix (ECM) proteins comparing starting tissue to tissues cultured for 14 days. n=3-11 (mean ± SEM), statistical differences were evaluated using one-way ANOVA with Sidak's multiple comparisons testing.

Supplementary Table 1

	**Uninfected**	**Convalescent**
**Tissues collected**	8/2020-07/2022	10/2020-07/2022
**Number collected**	10	8
**Median Age (Range)**	62.5 (48-76)	60.5 (40-73)
**Male**	4/10 (40%)	4/8 (50%)
**Ethnicity**		
Caucasion	9/10 (90%)	5/8 (62.5%)
Black	1/10 (10%)	3/8 (37.5%)
Average Convalescence Period (Range)		~191.6 Days (28-450 days)*
Vaccinated	2/10 (20%)	4/8 (50%)

Supplementary Table 1: Patient Demographics.

Supplementary Table 2

COVID-19+ Samples				
Sample	Convalescenece Period Before Resection	Sex	Vaccination Status	Other
5	28 days	Female	Not Vaccinated	
8	77 days	Male	Not Vaccinated	
10	104 days	Female	Vaccinated	First dose only; 27 days before resection
11	75 days	Male	Not Vaccinated	Tested positive twice (~2 and 9 months prior to resection)
13	450 days	Female	Vaccinated	Vaccinated (two doses) post COVID, ~1 year prior to tissue resection
15	450 days	Male	Vaccinated	Vaccinated (two doses) post COVID ~1 year prior to tissue resection, boosted ~ 6 months prior to tissue resection
17	148 days	Female	Vaccinated	Vaccinated (2 doses) ~9 months before resection, boosted ~5 months before resection
18	201 days	Male	Not Vaccinated	

Supplementary Table 2: COVID-19 Convalescent Extended Demographics.

Supplementary Figure 2

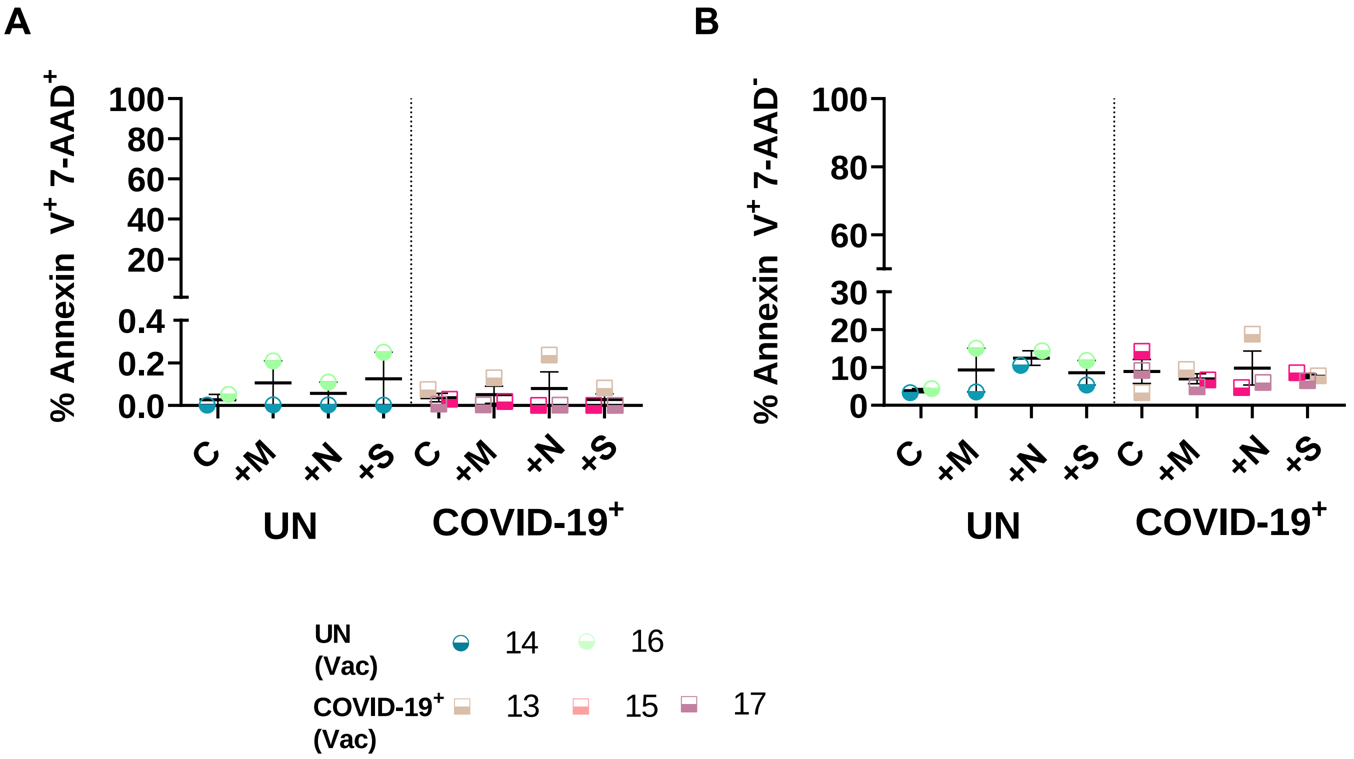

Supplementary Figure 2: Tissue Viability during 6 Day *Ex Vivo* Culture. **A.** Percentage of late apoptotic/necrotic cells following 6 days *ex vivo* culture. **B.** Percentage of early apoptotic cells following 6 days *ex vivo* culture. n=2 UN and n=3 COVID-19⁺ (mean (center line) ± SEM).

Supplementary Figure 3

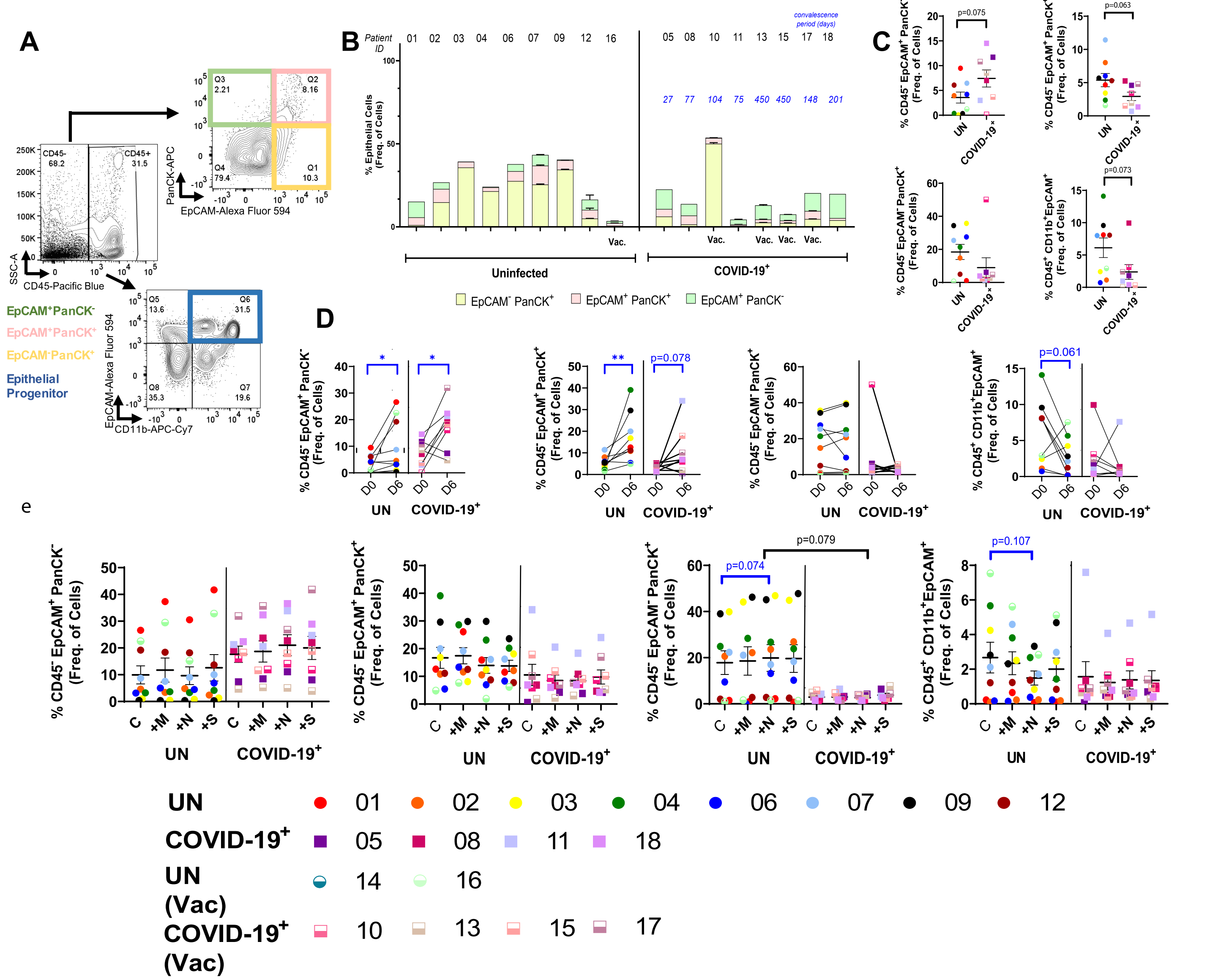

Supplementary Figure 3: Epithelial Cell Population Characterization following SARS-CoV-2 Peptide Exposure. **A**. Gating strategy for epithelial cells. **B**. Proportion of epithelial cell populations in starting tissues of UN and COVID-19⁺ lung tissues. **C**. Baseline differences in epithelial cell populations within UN and COVID-19⁺ lung tissues. **D**. Changes in epithelial cell populations over the culture period (day 0 vs. day 6 control). **E**. Impact of SARS-CoV-2 peptide exposure on epithelial cell populations in UN and COVID-19⁺ lung tissue. n=10 UN and n=8 COVID-19⁺ (mean (center line) ± SEM). Statistics shown in blue are comparisons between control and peptide exposed samples within each group (UN and COVID-19⁺). Statistics shown in black are the change in response between UN and COVID-19⁺ for each peptide when compared to the corresponding controls.

Supplementary Figure 4

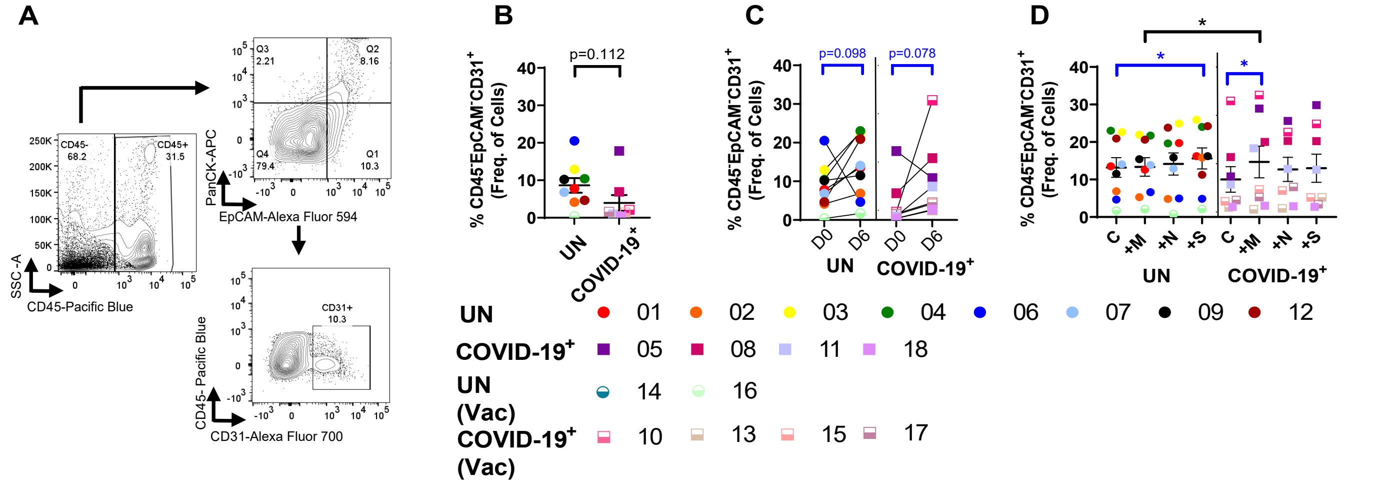

Supplementary Figure 4: Endothelial Cell Characterization with SARS-CoV-2 Peptide Exposure. **A.** Gating strategy for endothelial cells. **B.** Baseline differences in endothelial cells within UN and COVID-19$^+$ lung tissues. **C.** Changes in endothelial cells over the culture period (day 0 vs. day 6 control). **D.** Impact of SARS-CoV-2 peptides on endothelial cells in UN and COVID-19$^+$ lung tissue. n=10 UN and n=8 COVID-19$^+$ (mean (center line) ± SEM). Statistics shown in blue are comparisons between control and peptide exposed samples within each group (UN and COVID-19$^+$). Statistics shown in black are the change in response between UN and COVID-19$^+$ for each peptide when compared to the corresponding controls.

Supplementary Figure 5

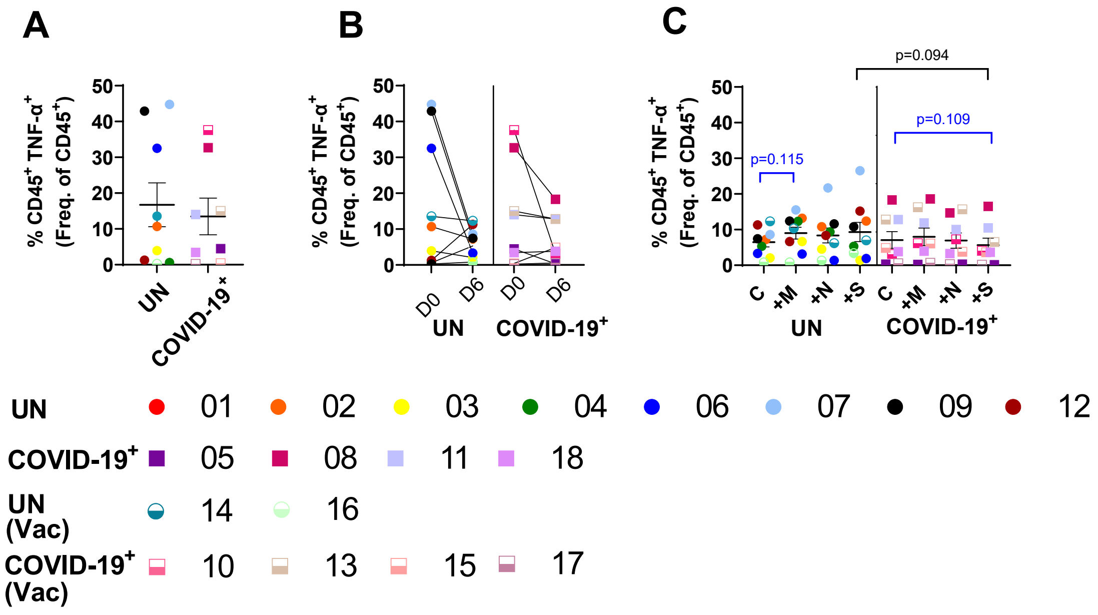

Supplementary Figure 5: Impact of SARS-CoV-2 Peptide Exposure on TNF-α-producing immune cells. **A**. Baseline differences in TNF-α⁺ immune cells within UN and COVID-19⁺ lung tissues. **B**. Changes in TNF-α⁺ immune cells over the culture period (day 0 vs. day 6 control). **C**. Impact of SARS-CoV-2 peptides on TNF-α⁺ immune cells in UN and COVID-19⁺ lung tissue. n=10 UN and n=8 COVID-19⁺ (mean (center line) ± SEM).

Supplementary Figure 6

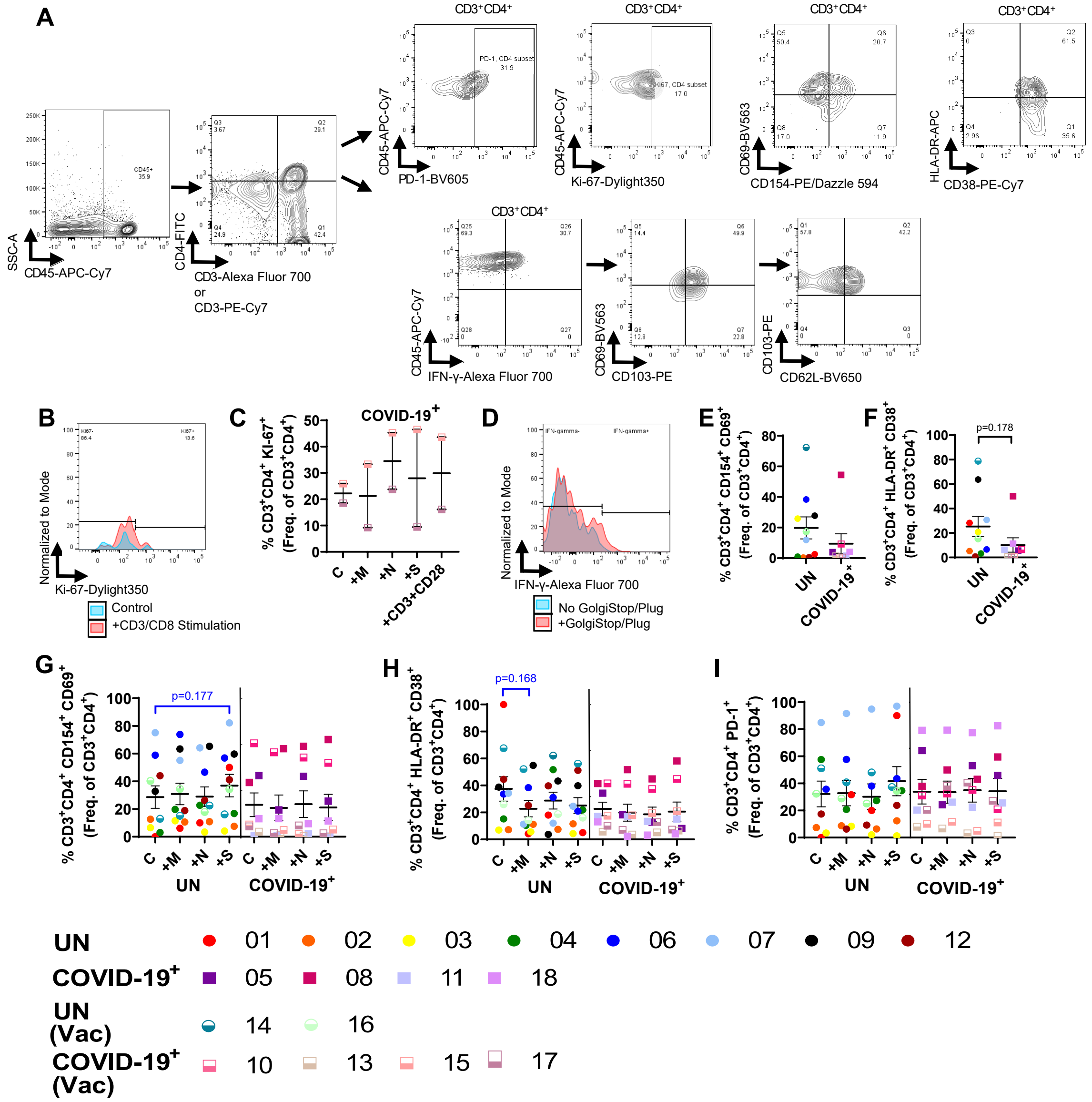

Supplementary Figure 6: Antigen-specific and Hyper-activated CD4$^+$ T cell changes in response to SARS-CoV-2 Peptide Exposure. **A**. Gating strategy for CD4$^+$ T cells. **B**. Histogram comparing Ki-67$^+$ CD4$^+$ T cells with CD3/CD28 stimulation and control tissue following 6 days culture. **C**. Ki-67$^+$ CD4$^+$ T cells with peptide stimulation or CD3/CD28 stimulation. **D**. Histogram comparing interferon-γ producing CD4$^+$ T cells with and without the inclusion of GolgiStop and GolgiPlug during peptide stimulation. **E-F**. Baseline differences in antigen-specific (**E**) and hyper-activated (**F**) CD4$^+$ T cell populations within UN and COVID-19$^+$ lung tissues. **G-I**. Impact of SARS- antigen-specific (**G**), hyper-activated (**H**), and PD-1$^+$ (**I**) CD4$^+$ T cell populations in UN and COVID-19$^+$ lung tissue. n=10 UN and n=8 COVID-19$^+$ (mean (center line) ± SEM). Statistics shown in blue are comparisons between control and peptide exposed samples within each group (UN and COVID-19$^+$). Statistics shown in black are the change in response between UN and COVID-19$^+$ for each peptide when compared to the corresponding controls.

Supplementary Figure 7

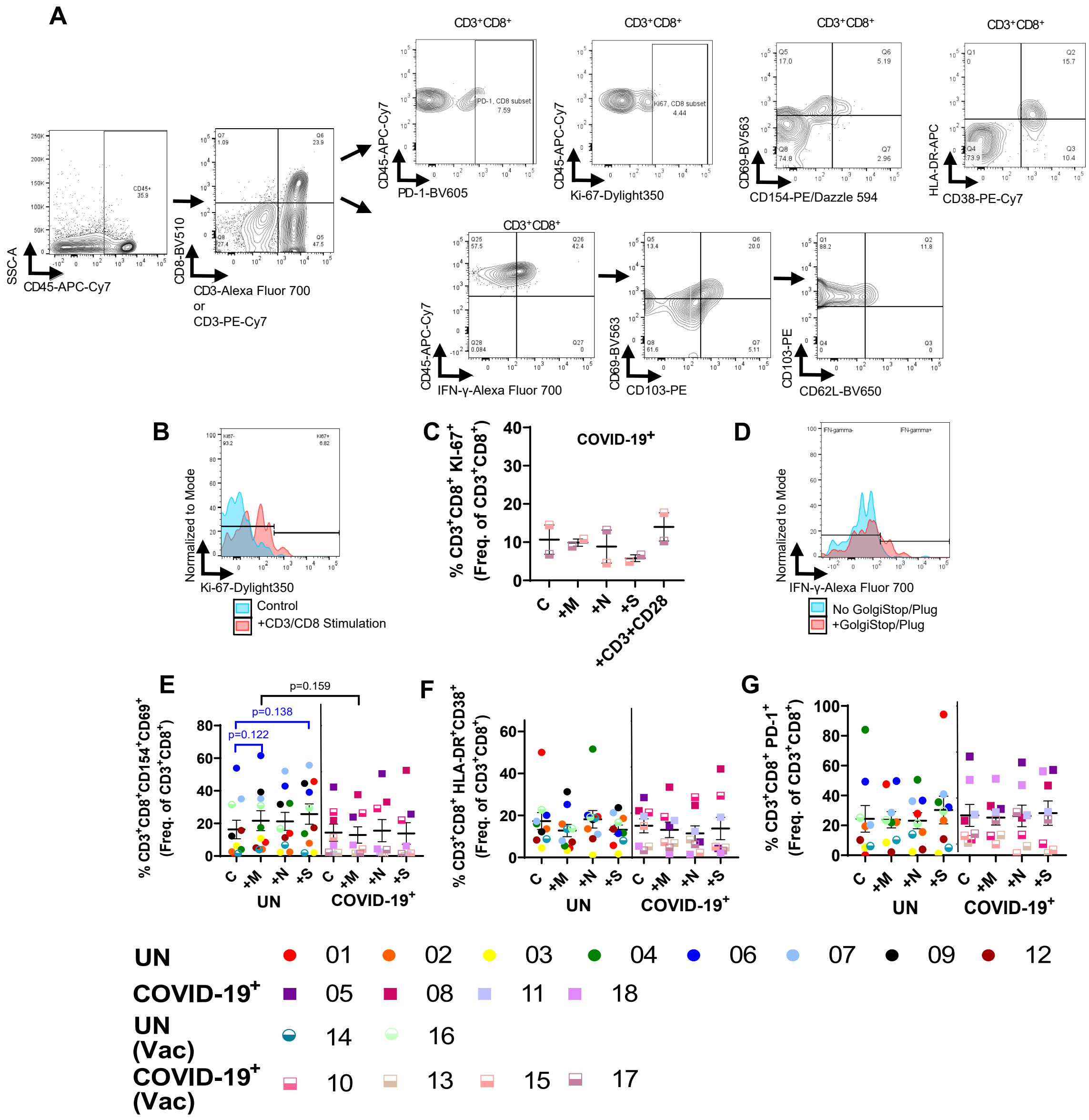

Supplementary Figure 7: Antigen-specific and Hyper-activated CD8$^+$ T cell changes in response to SARS-CoV-2 Peptide Exposure. **A**. Gating strategy for CD8$^+$ T cells. **B**. Histogram comparing Ki-67$^+$ CD8$^+$ T cells with CD3/CD28 stimulation and control tissue following 6 days culture. **C**. Ki-67$^+$ CD8$^+$ T cells with peptide stimulation or CD3/CD28 stimulation. **D**. Histogram comparing interferon-γ producing CD8$^+$ T cells with and without the inclusion of GolgiStop and GolgiPlug during peptide stimulation. **E-G**. Impact of SARS-CoV-2 peptide exposure on antigen-specific (**E**), hyper-activated (**F**) and PD1$^+$ (**G**) CD8$^+$ T cell populations in UN and COVID-19$^+$ lung tissue. n=10 UN and n=8 COVID-19 $^+$ (mean (center line) ± SEM). Statistics shown in blue are comparisons between control and peptide exposed samples within each group (UN and COVID-19$^+$). Statistics shown in black are the change in response between UN and COVID-19$^+$ for each peptide when compared to the corresponding controls.

Supplementary Figure 8

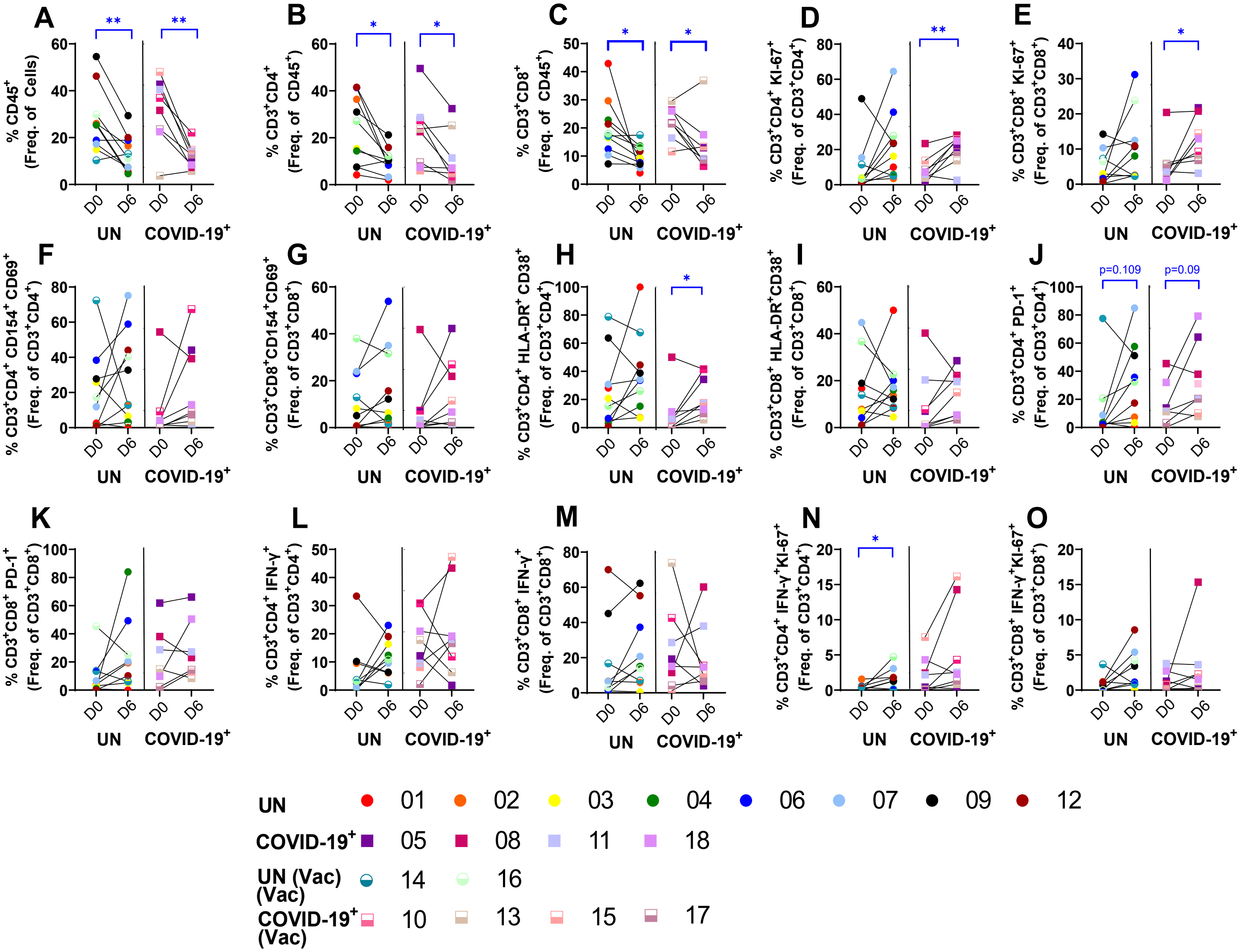

Supplementary Figure 8: T Cell Population Changes over the Culture Period. **A-O**. Changes in T cell populations over the culture period (day 0 vs. day 6 control). n=10 UN and n=8 COVID-19⁺ (mean (center line) ± SEM). Statistics shown in blue are comparisons between control and peptide exposed samples within each group (UN and COVID-19⁺). Statistics shown in black are the change in response between UN and COVID-19⁺ for each peptide when compared to the corresponding controls.

Supplementary Figure 9

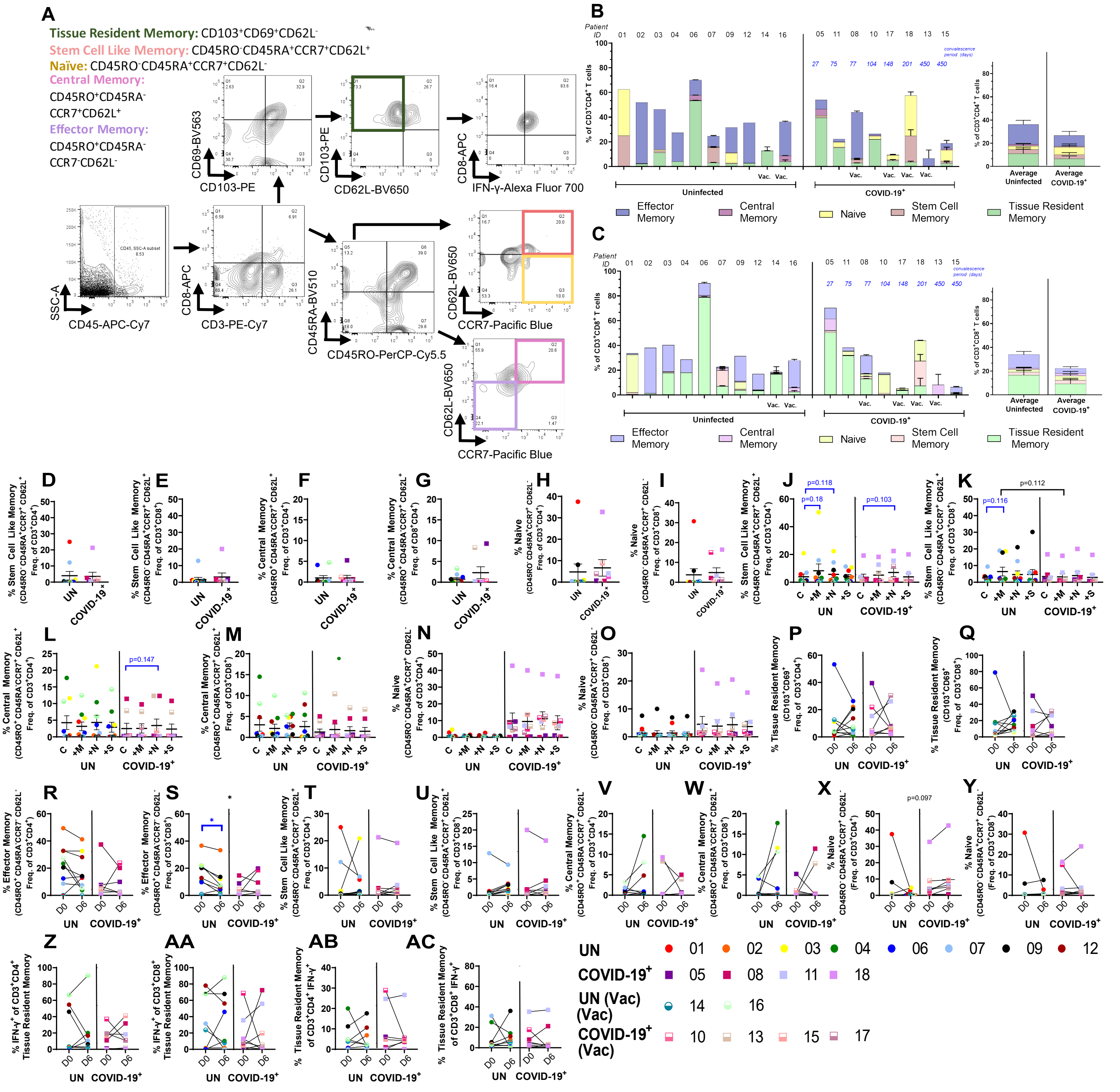

Supplementary Figure 9: Gating Strategy for Memory T cells, Starting Populations, and Changes over the Culture Period. **A**. Gating strategy for Memory T cells. **B-C**. Proportion of memory CD4⁺ (**B**) and CD8⁺ (**C**) T cell populations in starting tissues of UN and COVID-19⁺ lung tissues (left: individual tissue specimen; right: average across sample group). **D-I**. Baseline differences in stem cell like memory T cells (**D & E**), central memory T cells (**F & G**), and naïve T cells (**H & I**) within lung tissue from UN and COVID-19⁺. **J-O**. Impact of SARS-CoV-2 peptide exposure on memory T cell populations in UN and COVID-19⁺ lung tissues. **P-AC**. Changes in memory T cell populations over the culture period (day 0 vs. day 6 control). n=10 UN and n=8 COVID-19⁺ (mean (center line) ± SEM). Statistics shown in blue are comparisons between control and peptide exposed samples within each group (UN and COVID-19⁺). Statistics shown in black are the change in response between UN and COVID-19⁺ for each peptide when compared to the corresponding controls.

Supplementary Table 3

CD8+CD3+TRM+IFNgamma+ (Freq. of CD8+CD3+TRM)								
**Group**	**Patient Number**	**Day 0**	**Control (day 6)**	**M peptide**	**N peptide**	**S peptide**	**Sex**	**Convalescence Period (Days)**
FEMALE								
**Convalescent**	5	2.42	0.55	1.74	0.55	0.69	Female	28
**Convalescent**	10	68.7	0	**84.6**	**50**	**50**	Female	104
**Convalescent**	17	9.086667	3.103333	5.6066667	3.96	3.323333	Female	148
**Convalescent**	13	0	5.566667	**33.333333**	32.5	8.453333	Female	450
MALE								
**Convalescent**	11	35.5	55.8	53.2	46.85	49.3	Male	75
**Convalescent**	8	6.016667	72.46667	54.4	34.7	66	Male	77
**Convalescent**	18	12.9	0	0	18.95	0	Male	201
**Convalescent**	15	4.766667	19.76667	16.666667	10.83333	0	Male	450

Spearman Correlation Coefficients, N = 8<br>Prob > |r| under H0: Rho=0				
	**Control**	**M**	**N**	**S**
**Control**	1.00000	0.39522<br>0.3325	0.16767<br>0.6915	0.37952<br>0.3538
**M**	0.39522<br>0.3325	1.00000	**0.83333**<br>**0.0102**	**0.87427**<br>**0.0045**
**N**	0.16767<br>0.6915	**0.83333**<br>**0.0102**	1.00000	**0.73055**<br>**0.0396**
**S**	0.37952<br>0.3538	**0.87427**<br>**0.0045**	**0.73055**<br>**0.0396**	1.00000

Supplementary Table 3: Correlation of IFN-γ^+ CD8$^+$ TRM with Sex and Peptide Response.

Supplementary Figure 10

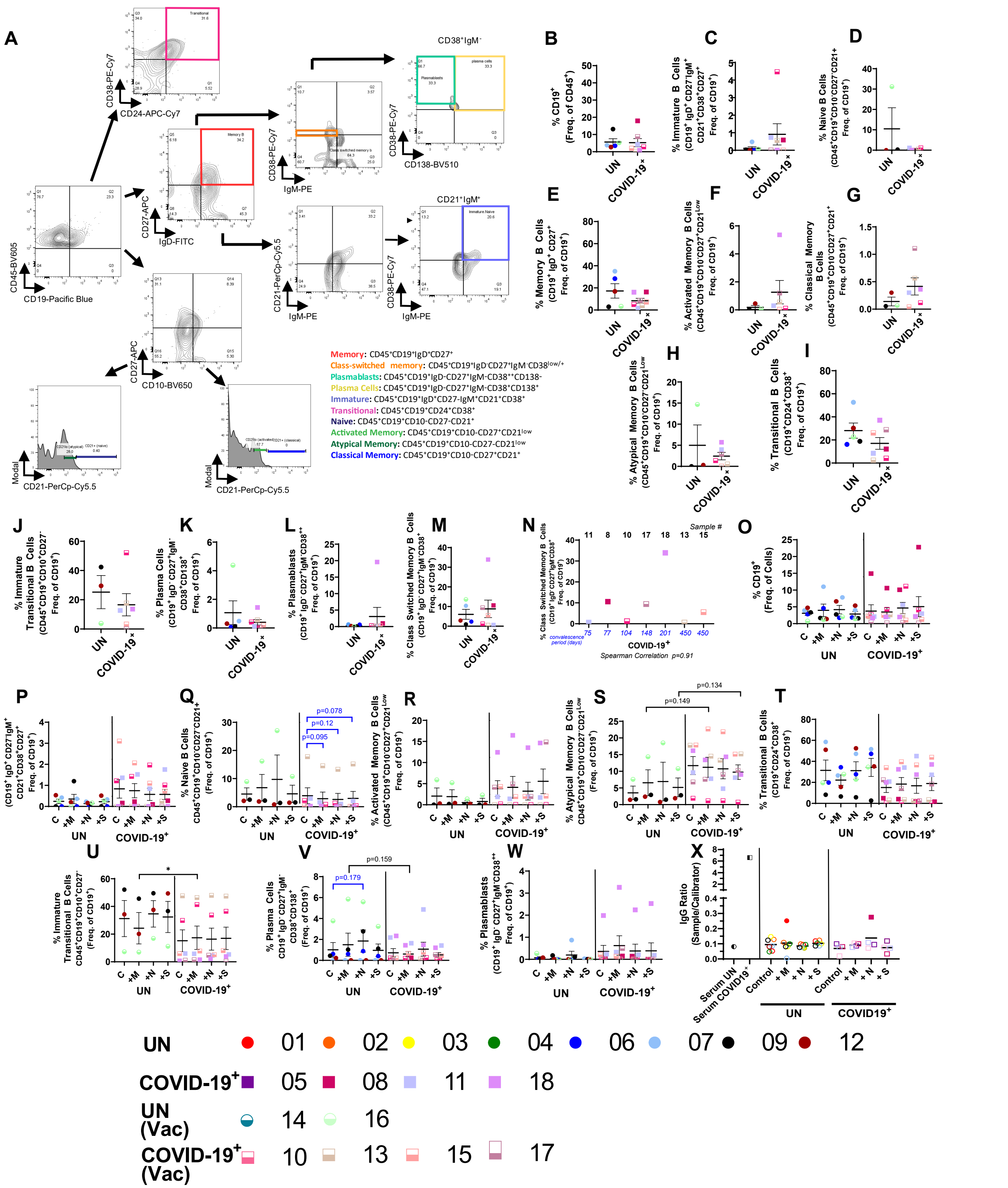

Supplementary Figure 10: Gating Strategy for B cells, Starting Populations, and Changes with Peptide Stimulation. **A**. Gating strategy for B cells. **B-M**. Changes in B cell populations in starting UN and COVID-19+ tissues. **N**. Correlation between convalescence period for COVID-19+ samples and percentage of Class Switched Memory B cells. **O-W**. Changes in B cell populations in UN and COVID-19+ tissues with peptide treatment. **X**. Changes in SARS-CoV-2 IgG with peptide treatment. n=5-10 UN and n=6-8 COVID-19+ (mean (center line) ± SEM). Statistics shown in blue are comparisons between control and peptide exposed samples within each group (UN and COVID-19+). Statistics shown in black are the change in response between UN and COVID-19+ for each peptide when compared to the corresponding controls.

Supplementary Table 4

Spearman Correlation: IgA Production & Class Switched Memory B cells

		Control (IgA)	convalescence period (IgA)	+ M peptide (IgA)	+ N peptide (IgA)	+ S peptide (IgA)	Control (class-switched mem. B)	Convalescence period (class-switched mem. B)	+M peptide (class-switched mem. B)	+N peptide (class-switched mem. B)	+S peptide (class-switched mem. B)
**Control (IgA)**	*Spearman Correlation Coefficients*	1	0.79043	0.78571	0.5	0.85714	0.21429	0.79282	0.28571	0.03571	0.21429
	Prob > |r| under H0: Rho=0		0.0195	0.0208	0.207	0.0065	0.6445	0.0334	0.5345	0.9394	0.6445
	Number of Observations	8	8	8	8	8	7	7	7	7	7
**Convalescence period (IgA)**	*Spearman Correlation Coefficients*	0.79043	1	0.85031	0.58684	0.82636	-0.12613	1	0.07207	-0.2883	0.07207
	Prob > |r| under H0: Rho=0	0.0195		0.0075	0.1262	0.0114	0.7876	<.0001	0.878	0.5307	0.878
	Number of Observations	8	8	8	8	8	7	7	7	7	7
**+ M peptide (IgA)**	*Spearman Correlation Coefficients*	0.78571	0.85031	1	0.80952	0.90476	-0.03571	0.91896	0.14286	-0.28571	0
	Prob > |r| under H0: Rho=0	0.0208	0.0075		0.0149	0.002	0.9394	0.0034	0.7599	0.5345	1
	Number of Observations	8	8	8	8	8	7	7	7	7	7
**+ N peptide (IgA)**	*Spearman Correlation Coefficients*	0.5	0.58684	0.80952	1	0.64286	0.07143	0.77481	0.21429	-0.07143	0.17857
	Prob > |r| under H0: Rho=0	0.207	0.1262	0.0149		0.0856	0.879	0.0408	0.6445	0.879	0.7017
	Number of Observations	8	8	8	8	8	7	7	7	7	7
**+ S peptide (IgA)**	*Spearman Correlation Coefficients*	0.85714	0.82636	0.90476	0.64286	1	0.10714	0.84688	0.28571	-0.10714	0.21429
	Prob > |r| under H0: Rho=0	0.0065	0.0114	0.002	0.0856		0.8192	0.0162	0.5345	0.8192	0.6445
	Number of Observations	8	8	8	8	8	7	7	7	7	7
**Control (class-switched mem. B)**	*Spearman Correlation Coefficients*	0.21429	-0.12613	-0.03571	0.07143	0.10714	1	-0.12613	0.96429	0.92857	0.82143
	Prob > |r| under H0: Rho=0	0.6445	0.7876	0.9394	0.879	0.8192		0.7876	0.0005	0.0025	0.0234
	Number of Observations	7	7	7	7	7	7	7	7	7	7
**Convalescence period (class-switched mem. B)**	*Spearman Correlation Coefficients*	0.79282	1	0.91896	0.77481	0.84688	-0.12613	1	0.07207	-0.2883	0.07207
	Prob > |r| under H0: Rho=0	0.0334	<.0001	0.0034	0.0408	0.0162	0.7876		0.878	0.5307	0.878
	Number of Observations	7	7	7	7	7	7	7	7	7	7
**+ M peptide (class-switched mem. B)**	*Spearman Correlation Coefficients*	0.28571	0.07207	0.14286	0.21429	0.28571	0.96429	0.07207	1	0.85714	0.85714
	Prob > |r| under H0: Rho=0	0.5345	0.878	0.7599	0.6445	0.5345	0.0005	0.878		0.0137	0.0137
	Number of Observations	7	7	7	7	7	7	7	7	7	7
**+ N peptide (class-switched mem. B)**	*Spearman Correlation Coefficients*	0.03571	-0.2883	-0.28571	-0.07143	-0.10714	0.92857	-0.2883	0.85714	1	0.89286
	Prob > |r| under H0: Rho=0	0.9394	0.5307	0.5345	0.879	0.8192	0.0025	0.5307	0.0137		0.0068
	Number of Observations	7	7	7	7	7	7	7	7	7	7
**+ S peptide (class-switched mem. B)**	*Spearman Correlation Coefficients*	0.21429	0.07207	0	0.17857	0.21429	0.82143	0.07207	0.85714	0.89286	1
	Prob > |r| under H0: Rho=0	0.6445	0.878	1	0.7017	0.6445	0.0234	0.878	0.0137	0.0068	
	Number of Observations	7	7	7	7	7	7	7	7	7	7

Supplementary Table 4: Correlation of IgA Production, Class Switched Memory B Cells, and Peptide Response.

Supplementary Figure 11

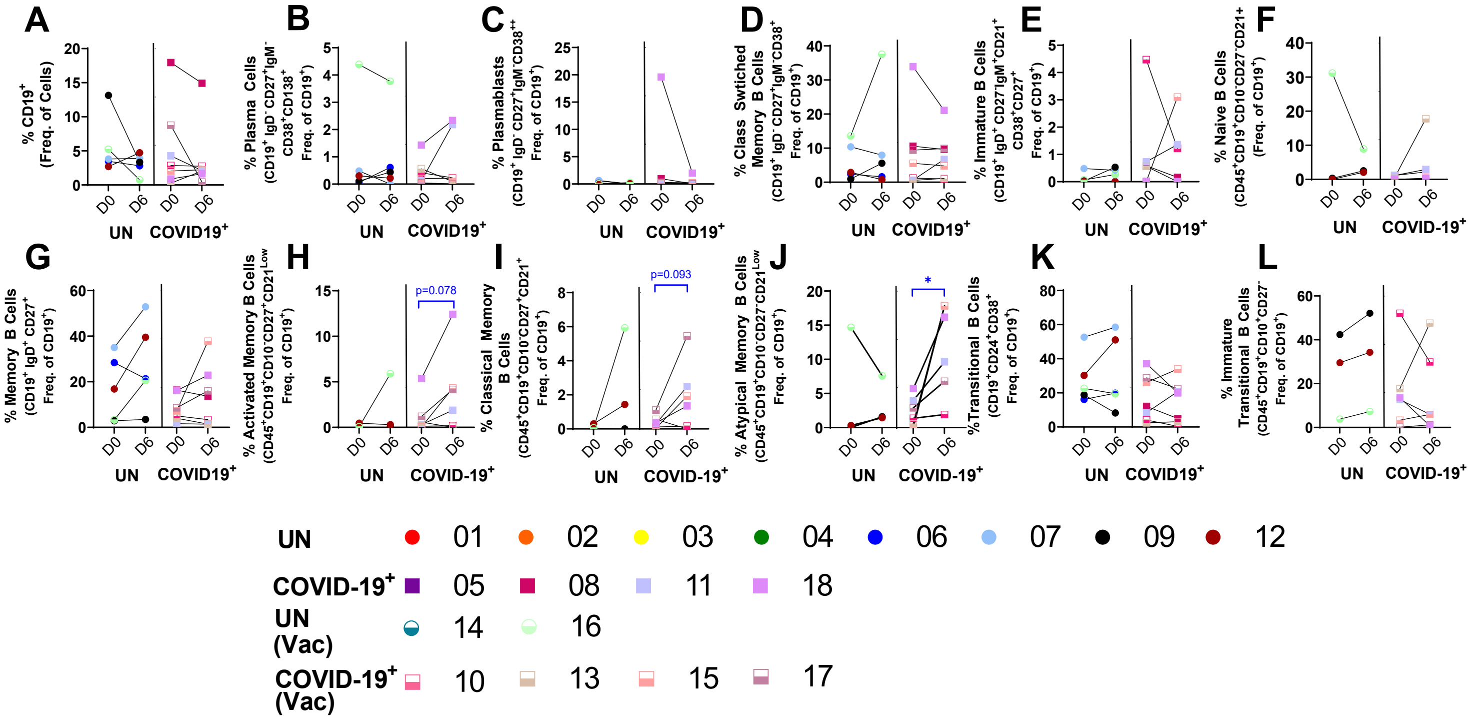

Supplementary Figure 11: B cell populations with culture. **A-L**. Changes in B cell populations over the culture period (day 0 vs. day 6 control). n=3-5 UN and n=6 COVID-19+ (mean (center line) ± SEM). Statistics shown in blue are comparisons between control and peptide exposed samples within each group (UN and COVID-19+). Statistics shown in black are the change in response between UN and COVID-19+ for each peptide when compared to the corresponding controls.

Supplementary Figure 12

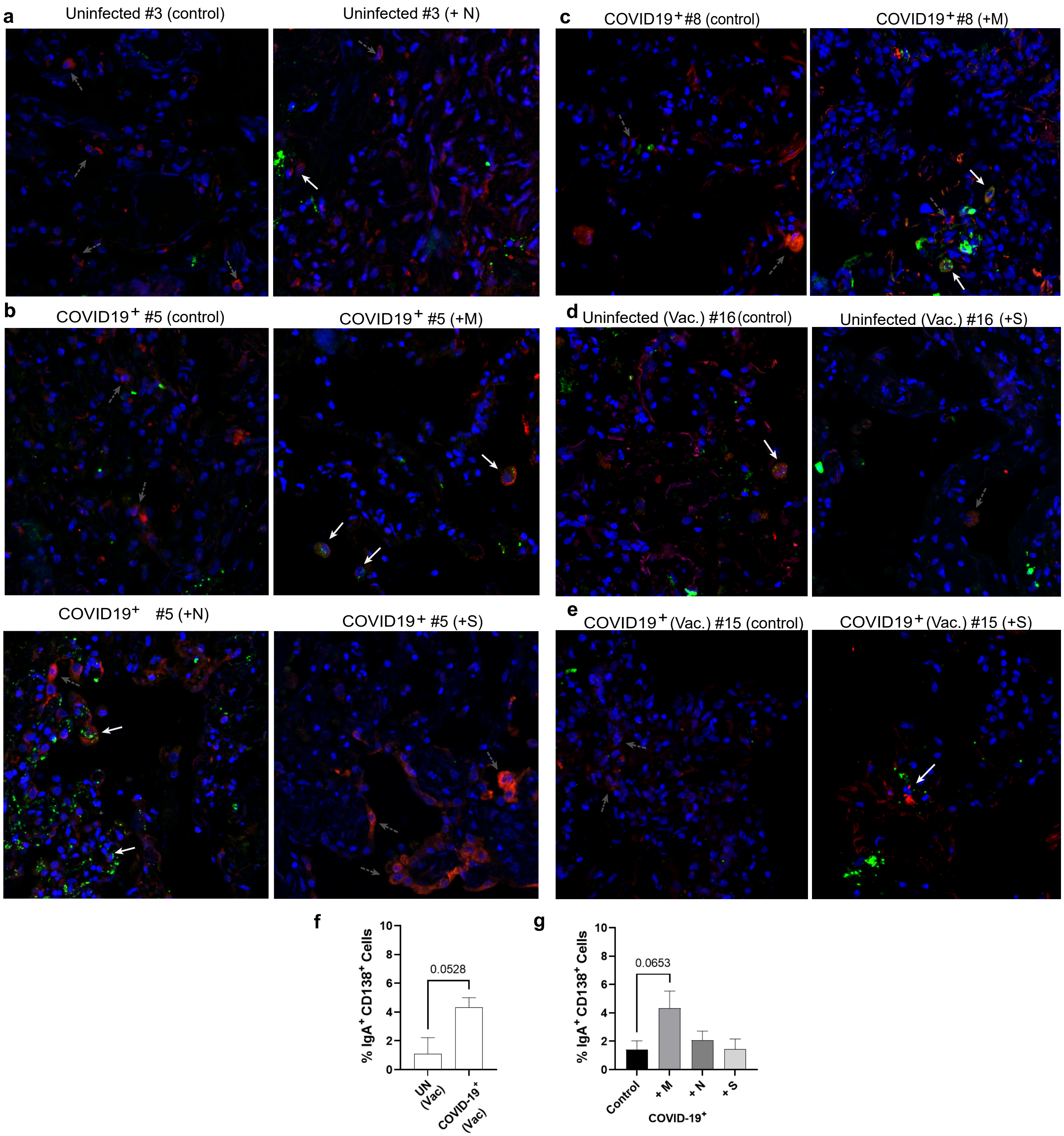

Supplementary Figure 12: CD138⁺ plasma cells within COVID-19⁺ samples secrete IgA in response to peptide stimulation. **A.** Photomicrographs of UN sample #3 showing CD138⁺ plasma cells (red) and IgA (green) in control tissue and in response to N peptide addition. **B-C.** Photomicrographs of COVID-19⁺ samples #5 and #8 showing CD138⁺ plasma cells and IgA in control tissue and in response to M, N, or S peptide addition. **D.** Photomicrographs of vaccinated UN sample #16 showing CD138⁺ plasma cells and IgA in control tissue and in response to S peptide addition. **E.** Photomicrographs of vaccinated COVID-19⁺ sample #15 showing CD138⁺ plasma cells and IgA in control tissue and in response to S peptide addition. White arrows pointing to CD138⁺ plasma cells secreting IgA, grey dashed arrows pointing to CD138⁺ plasma cells without IgA secretion. **F-G**. Quantification of proportion of IgA⁺ CD138⁺ cells at baseline (day 0, **F**) and with peptide stimulation in COVID-19⁺ samples (**G**).

Supplementary Figure 13

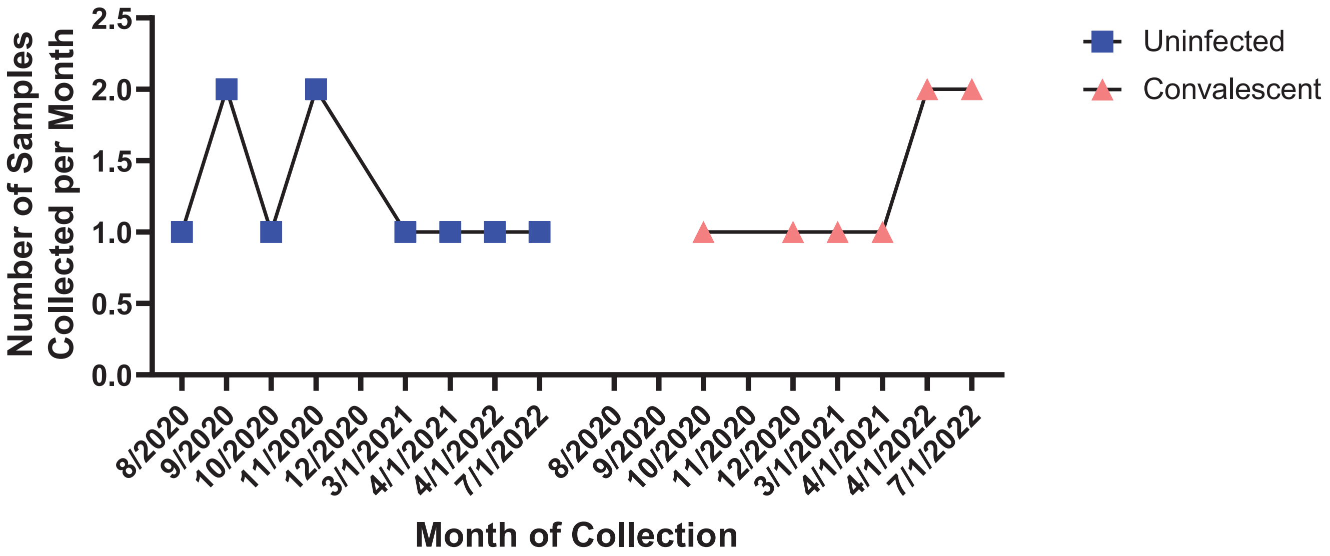

Supplementary Figure 13: Sample collection statistics.

Supplementary Figure 14

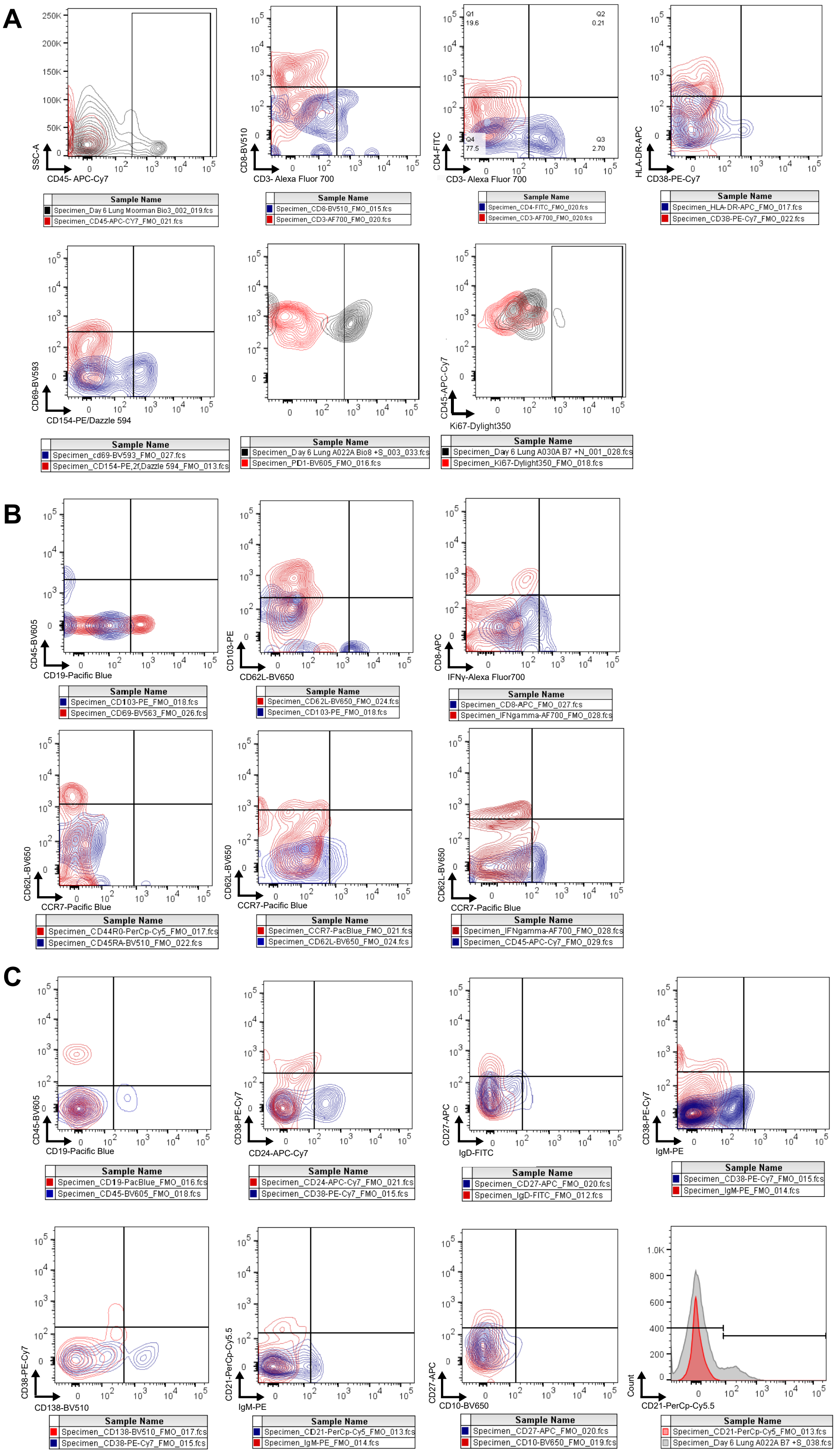

Supplementary Figure 14: FMO control plots showing T cell gating (**A**), memory T cell gating (**B**), and B cell gating (**C**).
